# Perinatal Morphine Exposure Leads to Sex-Dependent Executive Function Deficits and Microglial Changes in Mice

**DOI:** 10.1523/ENEURO.0238-22.2022

**Published:** 2022-10-13

**Authors:** Brittany L. Smith, Tess A. Guzman, Alexander H. Brendle, Collin J. Laaker, Alexis Ford, Adam R. Hiltz, Junfang Zhao, Kenneth D. R. Setchell, Teresa M. Reyes

**Affiliations:** 1 Department of Pharmacology & Systems Physiology, University of Cincinnati, Cincinnati, Ohio 45267; 2 College of Nursing, University of Cincinnati, Cincinnati, Ohio 45221; 3 Neuroscience Training Program, University of Wisconsin-Madison, Madison, Wisconsin 53705; 4 Clinical Mass Spectrometry Laboratory, Division of Pathology and Laboratory Medicine, Cincinnati Children's Hospital Medical Center, Cincinnati, Ohio 45229-3026; 5 Department of Pediatrics, University of Cincinnati College of Medicine, Cincinnati, Ohio 45267

**Keywords:** amygdala, executive function, microglia, prefrontal cortex, prenatal opioid, social behavior

## Abstract

Children exposed prenatally to opioids are at an increased risk for behavioral problems and executive function deficits. The prefrontal cortex (PFC) and amygdala (AMG) regulate executive function and social behavior and are sensitive to opioids prenatally. Opioids can bind to toll-like receptor 4 (TLR4) to activate microglia, which may be developmentally important for synaptic pruning. Therefore, we tested the effects of perinatal morphine exposure on executive function and social behavior in male and female mouse offspring, along with microglial-related and synaptic-related outcomes. Dams were injected once daily subcutaneously with saline (*n* = 8) or morphine (MO; 10 mg/kg; *n* = 12) throughout pregestation, gestation, and lactation until offspring were weaned on postnatal day 21 (P21). Male MO offspring had impairments in attention and accuracy in the five-choice serial reaction time task, while female MO offspring were less affected. Targeted gene expression analysis at P21 in the PFC identified alterations in microglial-related and TLR4-related genes, while immunohistochemical analysis in adult brains indicated decreased microglial Iba1 and phagocytic CD68 proteins in the PFC and AMG in males, but females had an increase. Further, both male and female MO offspring had increased social preference. Overall, these data demonstrate male vulnerability to executive function deficits in response to perinatal opioid exposure and evidence for disruptions in neuron–microglial signaling.

## Significance Statement

This study demonstrates (1) construct validity for a mouse model of perinatal morphine exposure that results in executive function deficits and (2) evidence in support of alterations in neuron–microglial signaling that may underlie these behavioral deficits. The importance of this work is emphasized by the continually worsening opioid epidemic and the challenges of studying long-term behavioral health of exposed children. Our study found that male morphine-exposed offspring had delayed learning and reduced motivation in basic cognitive tasks, followed by executive function deficits in attention and accuracy. Females instead had an increase in impulsivity, but both sexes displayed heightened social preference. Transcriptional profiling identified altered neuron–microglial signaling as an important biological pathway affected by perinatal morphine.

## Introduction

Prenatal opioid exposure rates are continuing to rise, largely captured by the 82% increase in Neonatal Opioid Withdrawal Syndrome (NOWS) incidence across the United States since 2010 (Hirai et al., 2021). As of 2017, NOWS rates were estimated at 7.3 per 1000 births, with some states seeing as high as 53.5 cases per 1000 births (Hirai et al., 2021) and males having an increased risk for NOWS diagnosis (Charles et al., 2017). Exposed infants have an increased risk for behavioral and emotional disorders, even in the absence of a NOWS diagnosis (Hall et al., 2019). Furthermore, exposed infants without NOWS are at a greater risk for increased mortality in the first year of life, emphasizing the need to study prenatal exposure even in the absence of NOWS (Leyenaar et al., 2021). While exposure is more difficult to quantify than a NOWS diagnosis, universal maternal drug testing suggests that prenatal opioid exposure could be as high as 10 times the national NOWS diagnosis rate (Smith et al., 2022a).

Children born to mothers taking opioid drugs during pregnancy have an increased risk for cognitive problems that most frequently manifest as executive function deficits during early childhood, including inattention and impulsivity (Hickey et al., 1995; Ornoy et al., 1996; Slinning, 2004; Wahlsten and Sarman, 2013; Nygaard et al., 2016; Levine and Woodward, 2018). These cognitive problems persist into adolescence (Nygaard et al., 2017), and males appear more vulnerable than females (Hickey et al., 1995; Nygaard et al., 2015). However, the complexities of the maternal opioid-using population (e.g., a high rate of polysubstance use; Smith, 2021) make it difficult to ascribe specific deficits to opioid exposure alone. This emphasizes the importance of animal models, and indeed, rodent studies have found cognitive deficits after prenatal opioid exposure (Zagon et al., 1979; Šlamberová et al., 2001; Wang and Han, 2009; Lu et al., 2012). Recently, adult rat offspring exposed to methadone during late gestation and lactation displayed specific executive function deficits in cognitive flexibility, but this was not assessed by sex (Jantzie et al., 2020).

Opioid maintenance therapy with methadone or buprenorphine is recommended for pregnant people who disclose an opioid use disorder, but major barriers can prevent access to these therapies (Mancher and Leshner, 2019). Hence, morphine (MO) remains among the most prevalent opioid detected (Smith et al., 2022a), likely since it is the immediate metabolite of heroin and a metabolite of codeine. Furthermore, morphine is frequently detected in mothers who tested positive for methadone or buprenorphine (Smith et al., 2022a).

Morphine and other opioids can activate toll-like receptor 4 (TLR4; Hutchinson et al., 2010; Wang et al., 2012), a pattern recognition receptor expressed primarily (but not exclusively) on microglia that initiates a cascade of cytokine release. In conjunction with deficits in executive function, methadone-exposed offspring (not differentiated by sex) were reported to have increased expression of TLR4 pathway genes in the cortex, along with increased IL-1β cytokine and Cxcl2 chemokine protein levels (Jantzie et al., 2020). Given this evidence and the known role of microglia in neurodevelopment (Paolicelli et al., 2011; Schafer et al., 2012; Zhan et al., 2014; Frost and Schafer, 2016; Kopec et al., 2018), it is possible that prenatal morphine exposure may impair executive function via altered microglial-mediated synaptic pruning. Furthermore, because microglial-mediated developmental processes differ substantially in males and females (Lenz et al., 2013; Lenz and McCarthy, 2015; McCarthy et al., 2017; Kopec et al., 2018; VanRyzin et al., 2019), the effects of prenatal morphine likely vary by sex.

The prefrontal cortex (PFC) is the brain region largely responsible for executive functioning, has heavy bidirectional connections with the amygdala (AMG), and opioid receptors are densely expressed in both PFC and AMG (Mansour et al., 1987). Importantly, children exposed prenatally to opioids have increased PFC–AMG connectivity, a finding that persists into adolescence (Radhakrishnan et al., 2021). We propose that the PFC and AMG are specifically vulnerable to the effects of prenatal opioid exposure.

Therefore, we hypothesized that long-term prenatal morphine exposure causes offspring changes in PFC-mediated and AMG-mediated behaviors that could vary by sex and potentially align with changes in microglia and synaptic markers. To study this, we used a 7 week perinatal morphine model that encompassed the pregestational, gestational, and lactation periods in mice to include exposure that is equivalent to third trimester brain development in humans. We assessed the social behavior of male and female offspring in adolescence using the three-chambered social interaction test and adult executive function in the operant-based touchscreen five-choice serial reaction time task (5CSRTT). We then examined the offspring PFC and AMG for gene expression at postnatal day 21 (P21) and in adulthood, along with quantifying microglial protein expression of Iba1 and lysosomal phagocytic marker CD68 in adult offspring. Finally, we conducted gene enrichment analysis on transcriptional changes in the P21 PFC to identify pathway-related hypotheses for future work.

## Materials and Methods

### Animal subjects

All experimental subjects were B6D2F1/J hybrid mice bred in-house from sexually naive female C57BL/6J and male DBA/2J mice that arrived at 7 weeks of age and acclimated to the temperature-controlled and humidity-controlled vivarium for 1 week before the experiment. This breeding enhances genetic diversity to increase the generalizability of our findings. All mice were housed in standard polycarbonate cages containing corncob bedding with water and food available *ad libitum* (unless otherwise noted for behavior testing). The housing room had a 12 h light/dark cycle (lights on, 6:00 A.M.; lights off, 6:00 P.M.). Experimental procedures were all conducted in compliance with the National Institutes of Health *Guidelines for the Care and Use of Laboratory Animals* and approved by the University of Cincinnati Institutional Animal Care and Use Committee.

### Experimental design and maternal morphine exposure

**Figure 1.**
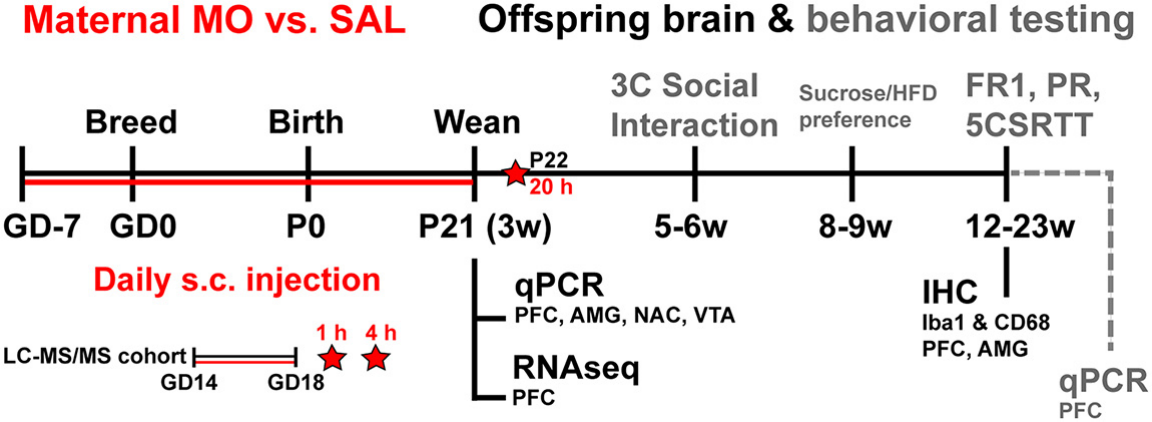
Experimental timeline. Perinatal morphine treatment to mouse dams (MO, 10 mg/kg, s.c.) versus SAL began 7 d before breeding [gestational day 7 (GD-7), start of the study], continued through birth and lactation until offspring were weaned on P21. Maternal plasma was collected on P22 for LC-MS/MS, 20 h after the final MO injection. One cohort of mouse offspring was used for P21 gene expression. A second behaviorally naive cohort was used for immunohistochemistry at 23 weeks (w) of age. The third cohort underwent testing for three-chambered social interaction, sucrose/high-fat diet preference, and operant testing (FR1; PR, progressive ratio), and brains were used for gene expression at 22 weeks. Finally, a smaller separate cohort of dams were injected at GD14–GD18, and maternal plasma, placentas, and fetal brains were collected at 1 and 4 h after MO injection for LC-MS/MS. Refer to Extended Data Figure 1-1 for sample sizes, and Extended Data Figures 1-2, 1-3, 1-4 for gene expression targets. Refer to Extended Data Figures 1-5, 1-6, 1-7 for additional birthing and offspring outcomes.

After 1 week acclimation to the facility, female C57BL/6J mice were randomly assigned to receive either MO (*n* = 12) or saline vehicle (SAL; *n* = 8), injected daily (subcutaneously), 4 h before lights off. Female injections began 1 week before breeding to habituate and minimize stress during pregnancy. Additionally, this models the establishment of opioid use before pregnancy, instead of after conception. Morphine sulfate (4 mg/ml stock; Henry Schein Medical Animal Health) was diluted with sterile saline to achieve an injection volume of 0.1–0.2 ml. Mice were injected with doses of 3, 5, and 7 mg/kg on days 1–3 to gradually acclimate the mice to the desired dose of 10 mg/kg on day 4, which was used throughout pregnancy and lactation, until offspring were weaned on P21 (Golalipour and Ghafari, 2012). Only dams were treated, offspring were not. Small litters were combined to ensure equal access to nutrition among pups, resulting in litter sizes of 8–10. After combining small litters together on P2 after the pup retrieval test (see below), the maternal sample size resulted in seven MO dams and six SAL dams. The dams that no longer had pups were removed from the study on P2. On P14, dams and their litters were moved to a room with a reversed light/dark cycle (lights off, 9:00 A.M.; lights on, 9:00 P.M.) to habituate offspring for behavioral testing during the dark cycle. The timing of the move was dictated by housing constraints within the vivarium, which habituation before breeding. On the day of weaning, one cohort of offspring were sacrificed on P21 for gene expression (*n* = 6/group; *n* = 1/litter/sex). The second cohort was used for all behavioral testing and adult gene expression and housed in cages of three to four, unless otherwise noted (*n* = 10–12/group; *n* = 1–2/litter/sex). Figure 1 shows the experimental timeline, and Extended Data Figure 1-1 shows a full description of sample sizes.

10.1523/ENEURO.0238-22.2022.f1-1 Figure 1-1 End point, variables measured, offspring sample size, and litter representation for each outcome assessed. Download Figure 1-1, DOCX file.

Dams used to breed the experimental cohort were killed 24 h after weaning offspring (20 h after the final 10 mg/kg, s.c., MO dose), and *n* = 2 plasma samples were quantified for MO concentrations. Additionally, a smaller separate cohort of dams was injected once daily for 5 d during the second gestational week. Dosing followed the same gradual increase across the first 4 d as used in the experimental dams: 3, 5, 7, 10 mg/kg. The 10 mg/kg dose was then administered again on the fifth day, and dams were killed 1 h (*n* = 2) or 4 h (*n* = 3) after injection. For dams from the experimental cohort killed at 20 h postinjection and the smaller cohort of dams killed at 1 and 4 h postinjection, maternal trunk blood was collected and centrifuged at 3500 × *g* for 15 min at 4°C. Fetal brains and placentas were collected from dams in the smaller cohort at 1 and 4 h (*n* = 3–4 offspring/dam) and flash frozen in isopentane. Plasma, brains, and placentas were stored at −80°C for mass spectrometry MO quantification. The small sample size used for this quantification was to verify MO transport to maternal plasma and fetal tissue, not for comparative purposes.

**Figure 2.**
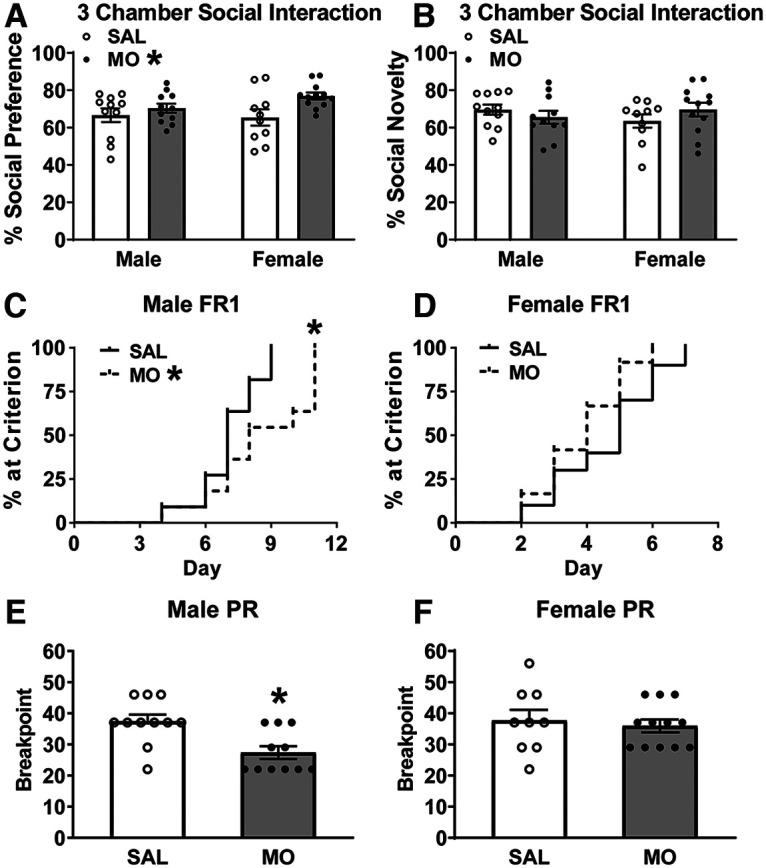
Social behavior and basic operant performance. ***A***, ***B***, Perinatal MO increased social preference (MO: *p < 0.05 main effect of MO vs SAL via two-way ANOVA; ***A***) but did not affect social novelty (***B***). ***C***, ***D***, Male MO offspring had delayed learning of FR1 (MO: *p < 0.05 vs SAL via Log rank survival analysis; ***C***), but not females (***D***). ***E***, ***F***, Male MO offspring had reduced break point in progressive ratio (PR; *p < 0.05 vs SAL via *t* test; ***E***), but not females (***F***). Refer to Extended Data Figure 2-1 for high-fat and sucrose preference test results.

10.1523/ENEURO.0238-22.2022.f1-2 Figure 1-2 Gene expression targets for P21 mPFC, AMG, VTA, and NAc. The + and – symbols indicate included (+) or omitted (–) targets for adult operant PFC. The ^ and ᵒ symbols indicate included (^) or omitted (ᵒ) targets for adult operant AMG. Download Figure 1-2, DOCX file.

**Figure 3.**
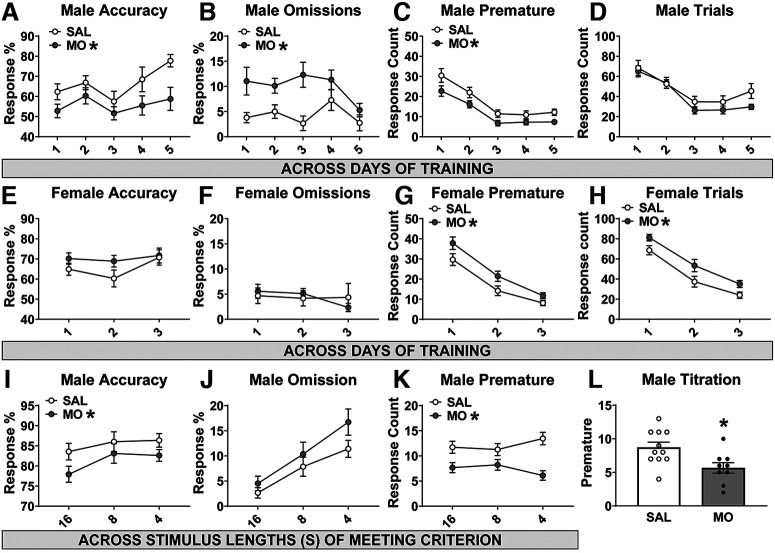
5CSRTT. ***A–D***, Male MO offspring had reduced accuracy (***A***), increased percentage of omissions (***B***), reduced number of premature responses (***C***), and no change in the total number of trials (***D***) in the initial training phase with a 16 s stimulus before reaching criterion (MO: *p < 0.05 main effect of MO vs SAL via two-way repeated-measures ANOVA). ***E–H***, Female MO offspring had no change in accuracy (***E***) or omissions (***F***), but had an increase in premature responses (***G***) and total number of trials (***H***) during 16 s stimulus training (MO: *p < 0.05 main effect of MO vs SAL via two-way repeated-measures ANOVA). ***I–K***, Upon successfully passing criterion at the 16, 8, and 4 s stimulus lengths, male MO offspring had reduced accuracy (***I***), no change in omissions (***J***), and reduced premature responses (***K***; MO: *p < 0.05 main effect of MO vs SAL via two-way repeated-measures ANOVA). ***L***, In the final titrated version of the 5CSRTT, male MO offspring had reduced premature responses (*p < 0.05 vs SAL, via *t* test).

10.1523/ENEURO.0238-22.2022.f1-3 Figure 1-3 Targets added for the adult operant mPFC gene expression analysis. The ^ and ᵒ symbols indicate included (^) or omitted (ᵒ) targets for adult operant AMG. Download Figure 1-3, DOCX file.

**Figure 4.**
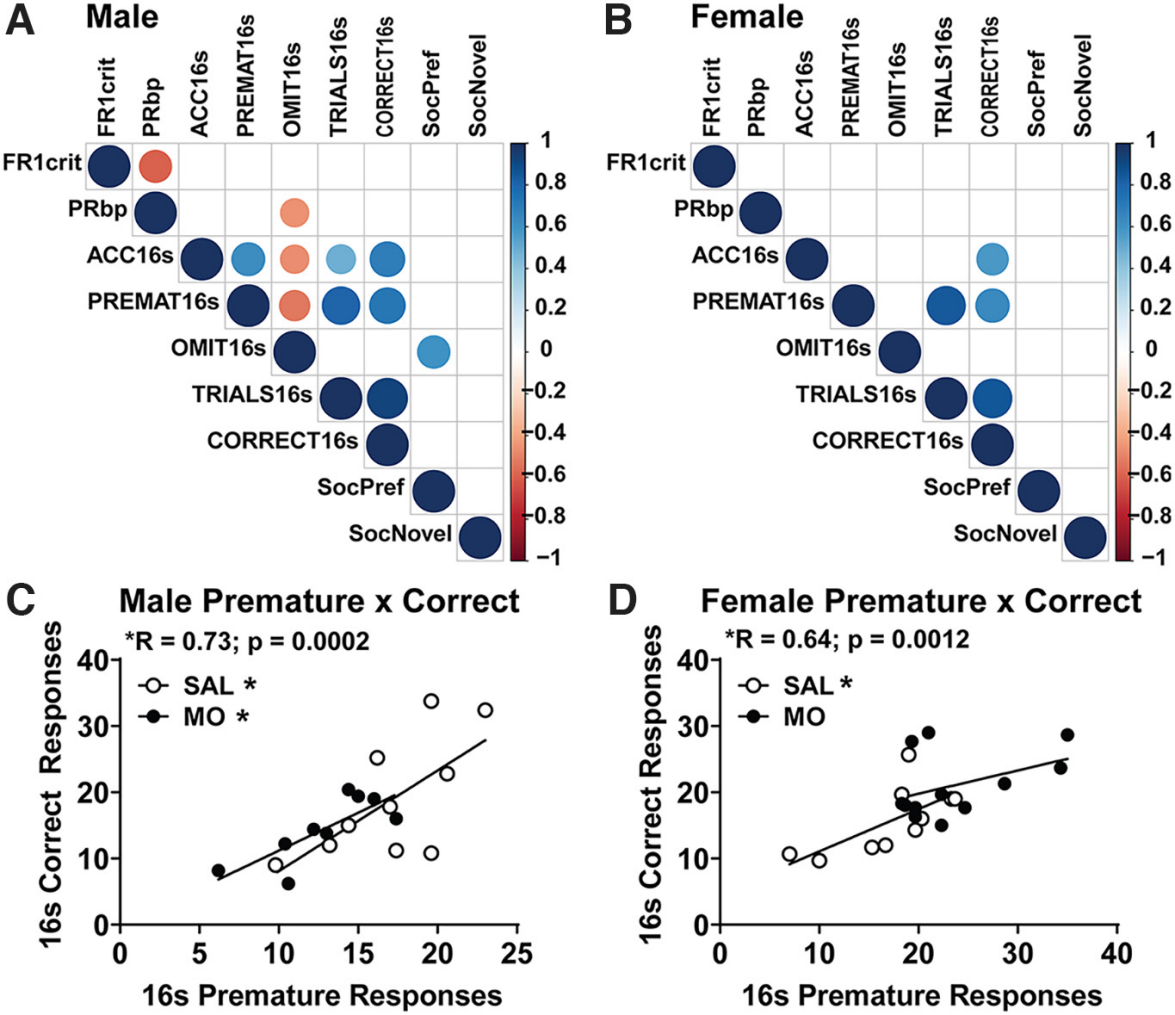
Behavioral correlations. ***A***, ***B***, Male (***A***) and female (***B***) correlation plots with circles denoting significant *p*-values (larger circle size showing smaller *p*-value) and the color of the circle showing the Pearson *r* value. ***C***, ***D***, Male (***C***) and female (***D***) linear regression plots depicting the correlation between premature and correct responses, further divided by MO versus SAL (*p < 0.05 via Pearson correlation). FR1 crit, Days to reach FR1 criterion; PRbp, break point in progressive ratio; ACC16s, average percentage accuracy (ACC) across initial 5CSRTT training with 16 s stimulus (16 s); PREMAT16s, average premature responses (16 s); OMIT16s, average percentage omissions (16 s); TRIALS16s, average trials (16s); CORRECT16s, average number of correct trials (16 s); SocPref, percentage preference for mouse versus empty cup in three-chamber social interaction test; SocNovel, percentage preference for novel versus familiar mouse in three-chamber social interaction test.

10.1523/ENEURO.0238-22.2022.f1-4 Figure 1-4 Targets added for the adult operant AMG gene expression analysis. Download Figure 1-4, DOCX file.

**Figure 5.**
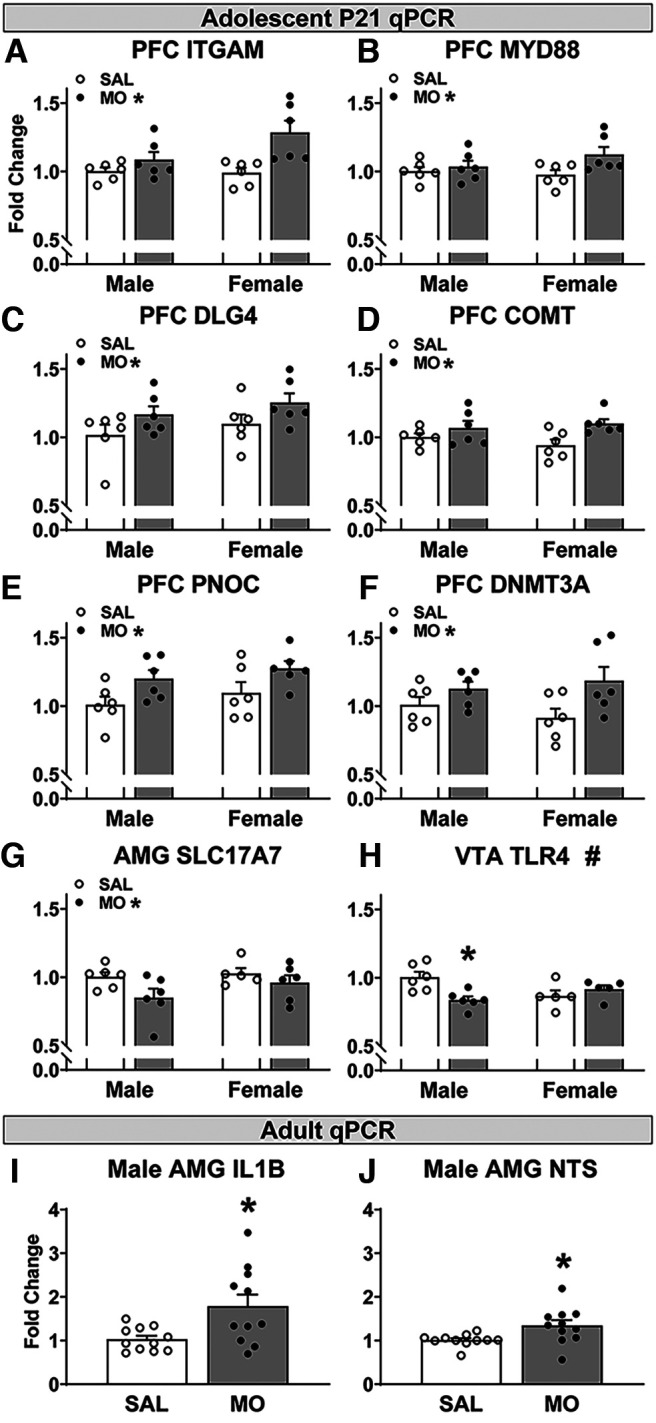
Postnatal day 21 and adult offspring gene expression (qPCR). ***A–F***, In the mPFC at P21, MO offspring had increased expression of ITGAM (CR3/CD11b; ***A***), MYD88 (***B***), DLG4 (PSD-95; ***C***), COMT (***D***), PNOC (***E***), and DNMT3A (***F***) (***A***-***F***, MO: *p < 0.05 drug main effect, two-way ANOVA). ***G***, In the AMG, MO offspring had reduced SLC17A7 (VGluT1) expression (MO: *p < 0.05 drug main effect, two-way ANOVA). ***H***, In the VTA, male MO had reduced TLR4 expression compares to male SAL (#*p* < 0.05 drug × sex interaction, *p < 0.05 vs male SAL, two-way ANOVA). ***I***, ***J***, In the adult AMG, male MO offspring had increased expression of IL-1β (***I***) and NTS (***J***). *p < 0.05 versus male SAL, *t* test. Refer to Extended Data Figure 5-1 for effects of sex at P21.

10.1523/ENEURO.0238-22.2022.f1-5 Figure 1-5 Maternal and birth characteristics. ***A***, ***B***, Maternal MO treatment did not alter gestational weight (***A***) or pup birth weight (***B***). ***C***, Assessed during the first postnatal week, MO injection acutely reduced time spent in the nest at 1 h, but this was recovered by 4 h (#*p* < 0.05, drug × time interaction; MO: *p < 0.05 main effect of drug; *p < 0.05 MO vs SAL at 1 h, two-way repeated-measures ANOVA). ***D***, On postnatal day 2, MO dams returned their pups faster on average than SAL dams (*p < 0.05 vs SAL, *t* test). Download Figure 1-5, TIF file.

**Figure 6.**
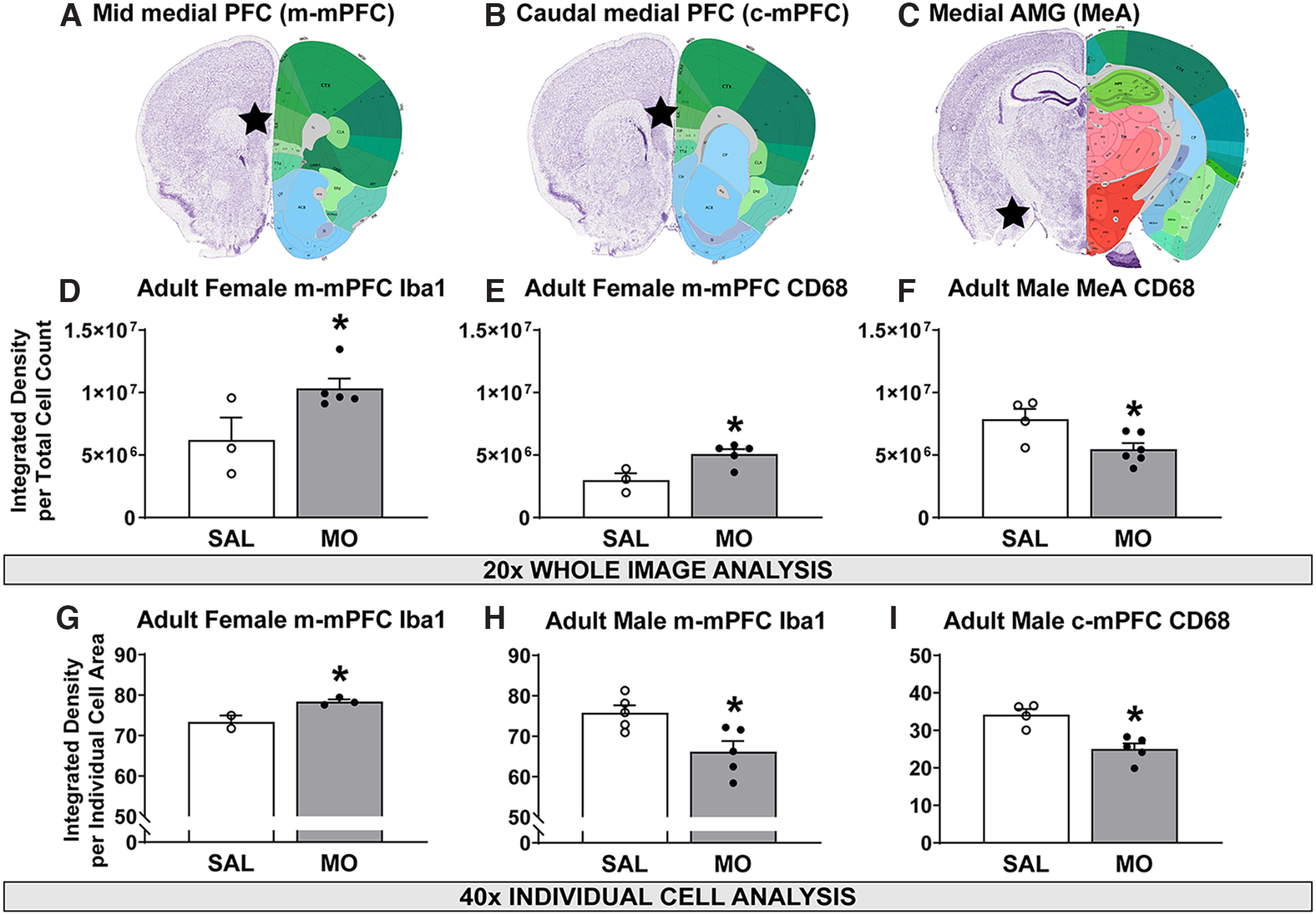
Adult Iba1 and CD68 immunohistochemistry. ***A–C***, Anatomical description of the m-mPFC (***A***), c-mPFC (***B***), and MeA (***C***). ***D***, ***E***, In 20× m-mPFC images, female MO offspring had increased Iba1 (***D***) and CD68 (***E***) integrated density normalized to cell count. ***F***, In 20× MeA images, male MO offspring had reduced CD68 integrated density normalized to cell count. ***G***, In 40× m-mPFC images, female MO offspring had increased Iba1 integrated density within the cell body. ***H***, ***I***, In 40× PFC images, male MO offspring had reduced Iba1 (***H***) and reduced CD68 integrated densities (***I***) within the cell body in the m-mPFC and c-mPFC, respectively. *p < 0.05 versus SAL, *t* test. Extended Data Figure 6-1 shows male m-mPFC values, and Extended Data Figure 6-2 shows representative immunohistochemical images.

10.1523/ENEURO.0238-22.2022.f1-6 Figure 1-6 Values for maternal and birthing outcomes that did not reach statistical significance (mean ± SEM) or median (range). Download Figure 1-6, DOCX file.

**Figure 7.**
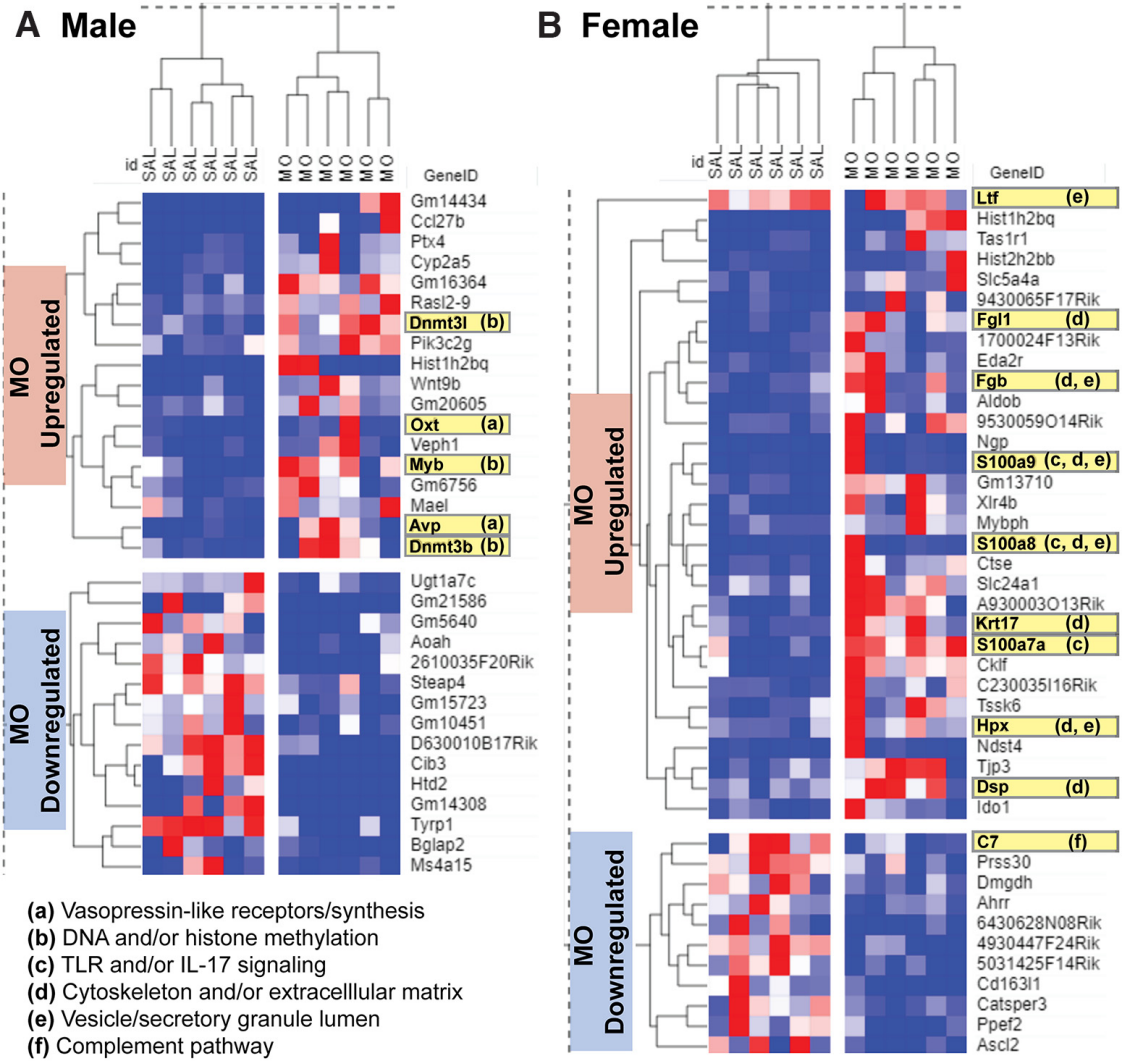
Postnatal day 21 mPFC exploratory RNA-seq. ***A***, ***B***, Male (***A***) and female (***B***) differentially expressed gene lists based on MO versus SAL comparison yielding unadjusted *p* < 0.05 and log2FoldChange >1.2 or less than −1.2. a–f, Genes highlighted in yellow depict contributions to significantly enriched pathways, annotated by functions. Hierarchical clustering gene × drug group dendrograms were computed with the Spearman rank-based similarity measure.

10.1523/ENEURO.0238-22.2022.f1-7 Figure 1-7 Values for offspring body weight (mean ± SEM). Download Figure 1-7, DOCX file.

### Pup retrieval test

On P2, the pup retrieval test was performed in the home cage during the light phase under baseline (BL) conditions (before daily injection). The dam was removed from the home cage and placed in a separate clean cage. The pups were then moved out of the nest and scattered apart from each other at the most distal portion of the home cage from the nest. The dam was then returned to the nest and video recorded undisturbed for 10 min. A single investigator blinded to the experimental groups used Behavioral Observation Research Interactive Software (BORIS) to quantify latency to retrieve the first pup, average retrieval latency per pup, and the percentage of pups retrieved during the 10 min session.

### Three-chambered social interaction test

At 5–6 weeks of age, adolescent offspring were tested in the three-chambered social interaction task (3C) during a single day under dim indirect white light. This was performed 1–8 h after lights off, with order balanced between groups. The apparatus (length, 52 cm; width, 26 cm; height, 22 cm) was divided into a central chamber (length, 14 cm) and two adjacent right and left chambers (length, 19 cm). Mice were first placed into the central chamber and allowed to explore all three chambers for 5 min (habituation). Next, mice were guided into the center chamber and briefly contained with dividers while a novel conspecific was placed into a wire cup on one of the side chambers. Dividers were then removed so the experimental mouse could explore for 10 min and become familiar with this mouse (social preference). Finally, the experimental mouse was brought back to the center chamber while a second novel conspecific was placed in the second cup, with the experimental mouse allowed to explore either mouse for 10 min (social novelty). The investigator counterbalanced the sides for familiar versus novel mice and whether the familiar mouse changed sides. Conspecific mice were B6D2F2/J hybrid mice from B6D2F1/J dams × DBA/2J males. A single investigator blinded to the experimental groups used BORIS to quantify time spent and bout frequencies investigating each of the cups under the various conditions (empty, mouse, familiar, novel). Preference and recognition indexes were calculated as percentages = [time spent investigating target/(time spent investigating target + time spent investigating other cup)] × 100.

### High-fat and sucrose preference tests

At 8–9 weeks of age, mice were singly housed and underwent preference testing for high-fat diet and sucrose (counterbalanced order on consecutive weeks). Mice were provided with either a 60% high-fat diet (catalog #58G9, TestDiet) or 4% sucrose water, in addition to standard chow diet and water bottles. Consumption of both options were measured daily for 3 d, with bottle/hopper placement switched daily. For both tests, day 1 was considered habituation and not assessed. Intake on days 2–3 was averaged and calculated as a percentage of total fluid or food intake. In the females, there were three SAL and three MO offspring that shredded the high-fat diet to a degree that prevented accurate measurement, resulting in seven SAL and nine MO mice. After completing both tests, mice were rehoused into their cages of three to four for the remainder of the study.

### Operant behavioral testing

At 12–13 weeks of age, adult offspring were food restricted to maintain 85–90% of their free-feeding body weight for 1 week before training and testing in Bussey-Saksida Mouse Touch Screen chambers using Abet II and Whisker software (Lafayette Instrument). Mice also habituated to the reinforcer for 24 h in their home cages (chocolate Yoo-hoo). Mice were tested 1×/d Monday–Friday for 30 min (or 1 h for progressive ratio) 1–8 h after lights off, with order balanced between groups.

#### Habituation, fixed ratio 1, and progressive ratio

Mice habituated to the chamber with magazine training for 3 d (5 Choice Habituation 2v2, Robbins). The habituation session began with a 5 s feeder pulse, and all remaining trials for the entire experiment were 280 ms. For magazine training, the magazine light illuminated until the mouse entered to collect the reward, followed by a 10 s intertrial interval (ITI). After habituation, the mice began fixed ratio 1 (FR1) training and testing, during which the center pad of five touchscreen pads became active (Center Only–Mouse Must Touch training v2). The center touchscreen illuminated until the mouse made a touch response, after which the magazine light illuminated until the mouse collected the reward, followed by a 1 s ITI. The lateral four touchscreen pads remained unlit, although touch responses to these blank pads were recorded. Achieving ≥70 trials for 2 consecutive days satisfied the FR1 learning criterion. Each male and female cohort remained on FR1 until all mice had passed the criterion. Once all mice had learned FR1 (7 d for females, 12 d for males), they underwent progressive ratio (PR) testing for 2 consecutive days. PR had the same stimulus and ITI as FR1, except after every three trials the number of responses needed to turn off the touchscreen and obtain a single reward increased in the following fashion: 1, 2, 4, 7, 11, 16, 22, 29, and 37. The break point was the response number achieved per reward at the end of the 1 h PR session.

#### Five-choice serial reaction time task

After PR testing, mice started the 5CSRTT, which began with 2 d of habituation to any one of the five touchscreen pads illuminating for a trial (5 Choice Mouse Must Touch, Robbins). The pad remained illuminated until the mouse made a correct response, followed by reward and 5 s ITI. All remaining 5CSRTT sessions had a finite stimulus presentation, and, for a successful trial, the mouse had to respond to the correct touch pad during illumination. Responding to an unlit pad during stimulus presentation counted as an incorrect trial, failure to respond at all during stimulus presentation counted as an omission, and any response during the 5 s ITI was a premature response. An incorrect, omitted, or premature response resulted in a 5 s time-out, during which the house light illuminated. All trials resulted in magazine light illumination, but correct responses also came with a 1 s tone and reward. The magazine light remained lit until the mouse entered to start the ITI. We analyzed the percentage of accuracy, the percentage of omissions, the total number of trials, and the total number of premature responses (Fletcher et al., 2007; Funk et al., 2019; Smith et al., 2020).

The mice completed four progressively harder 5CSRTT schedules at their own learning pace. A mouse passed schedule criterion by reaching at least 20 trials and >50% correct for 2 consecutive days. The first three schedules were finite stimulus presentations that remained constant over the 30 min session and lasted for 16, 8, and 4 s (Titration 5-Choice Mouse Touch Basic v3, Robbins; session values 10, 11, 12). For the 16 s stimulus, we analyzed the first few days of training the mice encountered before any mouse passed criterion. We then analyzed the first day that each mouse passed criterion for the 16, 8, and 4 s stimulus. After passing the 4 s stimulus, the mice underwent titration testing where stimuli durations were titrated to individual performance during the session (Martin et al., 2015; McKee et al., 2017). Correct trials decreased the stimulus duration by one level on the following trial, omissions or incorrect responses increased the stimulus duration by one level, while premature responses did not change stimulus duration. Titration sessions began with a 10 s stimulus, and the levels were 10, 8, 6, 4, 2, 1.5, 1, 0.9, 0.8, and 0.7 s until the mouse reached the minimum achievable stimulus. After 24 and 29 d of 5CSRTT in males and females, two male MO offspring and two female SAL offspring failed to pass the criteria to reach titration testing and were removed from analyses.

### Gene expression

One group of offspring was killed via rapid decapitation without perfusion on P21 and a second group in adulthood the morning after the final day of operant testing at 22 weeks of age. Male and female brains were promptly removed and immediately stored in RNAlater at 4°C overnight and at −20°C until dissection with a brain block to separate the medial PFC [mPFC; 1 mm sections; anteroposterior (AP), +1.2 to 2.2; dorsoventral (DV), −2.2 to 0.0; mediolateral (ML) ±0–0.6; Paxinos and Franklin, 2004] and AMG (2 mm sections; AP, −0.5 to 2.5; DV, −4 to 5.4; ML, ±1.6–3.8). From the P21 samples, the nucleus accumbens (NAc; 1 mm sections; AP, +1.2 to 2.2; DV, −2.2 to 3.6; ML, ±0–2.4) and ventral tegmental area (VTA; 1 mm gross sections; AP, −2.90 to 3.90; DV, −2.0 to 5.0; ML, ± 0–2.0) were also dissected. We included the NAc and VTA based on reports of increased reward-driven behavior in MO offspring (Ramsey et al., 1993; Hol et al., 1996; Grecco and Atwood, 2020). For RNA extraction, samples were homogenized in 500 μl of TRIzol reagent (Thermo Fisher Scientific) with a TissueLyser (Qiagen). After incubation with 100 μl of chloroform (20 min) and centrifugation (15 min, 12,000 × *g*, at 4°C), the supernatant was mixed with 100% ethanol, transferred to RNeasy columns, and washed with buffers RWT and RPE (Qiagen). An Epoch microplate spectrophotometer (BioTek) was used to assess RNA quantity and quality and cDNA was synthesized with a High Capacity Reverse Transcriptase Kit (Thermo Fisher Scientific). The cDNA samples were preamplified for 32 genes of interest, using TaqMan assays and Preamp Master Mix (Thermo Fisher Scientific) following the manufacturer protocol (Fluidigm) and as previously published (McKee et al., 2017; Smith et al., 2020). For quantitative PCR (qPCR), CT values were obtained for each of the 32 genes using a 96.96 Dynamic Array Integrated Fluidic Circuit with Biomark HD (Fluidigm). Extended Data Figure 1-2 shows P21 targets, Extended Data Figure 1-3 shows added adult mPFC targets, and Extended Data Figure 1-4 shows added adult AMG targets (the adult AMG samples were run on a plate with another study, for which targets were chosen separately). NormFinder was used to identify the most stable housekeeping gene pair for each brain region and age group from a list of four: ACTB, GAPDH, PPIA, YWHAZ. NormFinder revealed that ACTB and PPIA were the most stable housekeeping gene pair for the AMG, NAc, and VTA, while GAPDH and PPIA were the most stable combination in mPFC. The ddCT method was used to normalize the expression of the 28 targets to the geomean of the most stable housekeeping gene pair, with the male SAL serving as the reference group for sex × drug analysis, and male or female SAL serving as the same-sex reference group for adult operant gene expression analysis.

### P21 mPFC RNA sequencing, data analysis, and pathway analysis

RNA samples used for P21 mPFC Fluidigm gene expression were also used for RNA sequencing (seq). The data discussed in this publication have been deposited in the National Center for Biotechnology Information Gene Expression Omnibus (Edgar et al., 2002) and are accessible through GEO Series accession number GSE206032 (https://www.ncbi.nlm.nih.gov/geo/query/acc.cgi?acc=GSE206032). Directional poly A RNA-seq was performed via previously described protocols (Walsh et al., 2019; Rapp et al., 2020). Briefly, total RNA quality was analyzed for quality control (QC) with Bioanalyzer (Agilent). Sample RNA integrity number (input resistance) values ranged from 7.2 to 8.9. To obtain poly A RNA for library preparation, 1 μg of high-quality total RNA was input into NEBNext Poly(A) mRNA Magnetic Isolation Module (New England BioLabs). The poly A RNA was then enriched using the SMARTer Apollo automated NGS Library Preparation system (Takara Bio USA). For library preparation, NEBNext Ultra II Directional RNA Library Prep Kit (New England BioLabs) was used with PCR cycle number 8. Next, library QC and quantification was completed with real-time qPCR (NEBNext Library Quant Kit, New England BioLabs) and individually indexed libraries were pooled proportionally and sequenced using NextSeq 550 Sequencer (Illumina), with the sequencing setting of single read 1 × 85 bp to generate ∼25 million reads.

After sequencing, Illumina BaseSpace Sequence Hub automatically produced Fastq files for downstream analysis. BaseSpace app RNA-Seq Alignment version 2.0.2 and RNA-Seq Differential Expression app version 1.0.1 were used for standard bioinformatics analysis to identify differentially expressed genes. Reference genome *Mus musculus*/MM10 (RefSeq) was used to align reads under first-strand setting, with the analysis using STAR for alignment and Salmon for quantification. BAM and Transcriptome.BAM files were obtained from STAR, with Salmon then using the Transcriptome.BAM file to assign transcripts per million to genes and transcripts. The RNA-Seq Differential Expression app then used the alignment result for differential expression analysis of reference genes with DESeq2 to create differential expression reports in BaseSpace Hub.

Gene set enrichment analysis was performed using Enrichr (Chen et al., 2013; Kuleshov et al., 2016; Xie et al., 2021). Enrichment analysis was conducted on upregulated and downregulated gene lists in both male and female offspring (MO vs SAL: unadjusted *p* < 0.05; and log2FoldChange >1.2 or less than −1.2), to identify biologically relevant pathways underlying the gene lists. Then to determine the robustness of the upregulated/downregulated gene lists in determining perinatal MO versus SAL exposure, unbiased gene × drug hierarchical clustering was performed using Morpheus (Broad Institute). Pearson and Spearman similarity measures were assessed, along with both hierarchical and k-means clustering (*k* = 2).

### Immunohistochemistry

A subset of adult behaviorally naive SAL and MO offspring from the same pregnancy cohort were transcardially perfused with 1× PBS followed by 4% paraformaldehyde at 23 weeks of age (male SAL, *n* = 5; male MO, *n* = 6; female SAL; *n* = 3; female MO, *n* = 5). Perfused brains were collected and stored in 4% paraformaldehyde for 4 h, transferred to 30% sucrose overnight, sectioned on a microtome at 35 μm, and stored in cryoprotectant. For immunohistochemistry, sections were washed 5× 5 min in 1× PBS before transfer to blocking solution (0.2% bovine serum albumin, 0.4% Triton X-100, 4% normal goat serum). After 1 h of incubation in blocking solution, sections were incubated in the following primary antibodies diluted in blocking solution overnight at 4°C: rabbit anti-Iba1 (1:1000; catalog #019–19 741, Wako Chemicals USA); and rat anti-CD68 (1:500; catalog #MCA1957, BIO-RAD). Following primary antibody incubation, samples were washed with 1× PBS and incubated 1:500 in goat anti-rabbit cyanine3 and 1:500 in goat anti-rat 647 IgG for 1 h. Sections were washed, mounted to slides, and coverslipped with DABCO Mounting Media. Images were captured at 20× and 40× magnification using Axiovision 4.6 software (Zeiss) with Apotome Deconvolution. At 40×, *z*-stacks were captured, and subsets of five consecutive 1 μm images in the stack were compressed into projection images (LSM Image Browser) as previously described (Kopp et al., 2013; Smith et al., 2016). Medial PFC images were taken bilaterally at both mid-mPFC (m-mPFC) and caudal mPFC (c-mPFC) sections at the junction between the prelimbic and infralimbic divisions, due to reports of rostral–caudal differences in mPFC control of cognition and motivation (Kouneiher et al., 2009). We did not separate the prelimbic from the infralimbic mPFC because of the lack of differences in Iba1 expression between these subdivisions in previous work (Kopp et al., 2013). Bilateral AMG images were taken of the basolateral amygdala (BLA) and medial amygdala (MeA). ImageJ was used for all quantification. At 20×, whole-frame immunofluorescence was assessed by counting Iba1-positive cells and normalizing the automated integrated density measurement of CD68 and Iba1 immunofluorescence to the cell count in the frame. At 40×, cell-specific immunofluorescence was assessed by outlining individual Iba1-positive cell bodies with the freehand tool. ImageJ quantification for each cell included automatic measurement calculations of: Iba1 cell perimeter, area, Iba1 integrated density/individual cell area, and CD68 integrated density/individual cell area. At 40×, there were issues with focusing some of the slides for the *z*-stacks, resulting in a smaller sample size (male SAL, *n* = 5; male MO, *n* = 6; female SAL, *n* = 3; female MO, *n* = 5).

### Liquid chromatography tandem mass spectrometry

Total morphine concentration was measured in mouse plasma, fetal brain, and placenta by tandem mass spectrometry (MS/MS) using a validated clinical assay for the detection of drugs of abuse in human urine and umbilical cord tissue performed according to proprietary assays (standard operating procedures #Path.CMS 1060 and #Path.CMS 1066) in a College of American Pathologists-accredited and Clinical Laboratory Improvement Amendments-accredited laboratory. Briefly, plasma and homogenized brain and placenta were hydrolyzed with a β-glucuronidase enzyme to obtain unconjugated morphine in the presence of the stable isotopic-labeled internal standard, [^2^H_6_]morphine (morphine-D_6_), and then extracted by solid-phase extraction on a Strata-X-C strong cation exchange column (Phenomenex). Morphine was eluted with organic solvent and liquid chromatography tandem mass spectrometry (LC)-MS/MS analysis was performed using a LC20AD HPLC system (Shimadzu) coupled to a mass spectrometer (model QTRAP 4500, SCIEX). Chromatographic separation was achieved on a Kinetex 2.6 μm Phenyl-Hexyl LC column (50 × 4.6 mm; Phenomenex). A gradient mobile phase was used with a binary solvent system, which was ramped from 5% mobile phase B (methanol/0.1% formic acid) to 5% mobile phase A (water/10 mm ammonium formate) at a flow rate of 0.7 ml/min. The total run time was 7 min. The optimal signal for the morphine and morphine-D_6_ were achieved in positive ion mode with the use of the following instrument settings: ion spray voltage, 2500 V; source temperature, 650°C; curtain gas, 35 psi; ion source gas 1, 60 psi; and ion source gas 2, 50 psi. The ion transitions monitored were selected as mass/charge ratio (*m*/*z*) of 286.0 to >152.1 (quantifier ion) and *m*/*z* of 286.0 to >165.1 (qualifier ion) for morphine, and *m*/*z* of 292.1 to >152.1 for morphine-D_6_. Data acquisition on the mass spectrometer was controlled by Analyst 1.7.2 software (SCIEX). Data processing and quantification were performed with MultiQuant software version 3.0.3 (SCIEX).

### Statistics

Data were analyzed with GraphPad Prism 9 (GraphPad Software) and are displayed as the mean ± SEM. Maternal data were analyzed with unpaired *t* tests (MO vs SAL) or two-way repeated-measures ANOVAs (drug × time). Offspring P21 gene expression and behavior data collected before operant testing were analyzed by two-way ANOVAs (drug × sex). Offspring operant behavior data and adult gene expression were analyzed separately for each sex with unpaired *t* tests (MO vs SAL) or two-way repeated-measures ANOVAs (drug × time). Females completed operant training in far fewer days than the males, with testing and tissue collection occurring on different days than the males. Furthermore, the pronounced sex differences in operant training and performance may obscure the detection of the effects of maternal drug history. Data that failed equal variance assumptions were analyzed via nonparametric Mann–Whitney test. For the percentage of passing the behavioral criterion, data were analyzed via Log-rank test comparing the survival curves. For significant interactions in the two-way ANOVAs/repeated-measures ANOVAs, *post hoc* testing was conducted to assess the effect of the drug using Bonferroni’s multiple-comparisons correction. Behavioral correlations were conducted in R using the corrplot package (Wei et al., 2021) with Pearson’s correlation measure, and significant correlations of interest graphed in Prism. Refer to Table 1 with the result column indicating superscript letters for each statistical result presented in the Results.

**Table 1:** Statistical table

Result	Data structure	Type of test	Power: 95% CI, MO vs SAL
a	Normal distribution	Two-way ANOVA	−0.9729 to 1.565
b	Normal distribution	Two-way ANOVA	12.37–57.91
c	Normal distribution	*t* test	−42.15 to −2.805
d	Normal distribution	Two-way ANOVA	−13.95 to −1.238
e	Survival analysis	Log-rank (Mantel–Cox)	0.8219–4.835
f	Normal distribution	*t* test	−16.25 to −3.751
g	Normal distribution	One-way ANOVA	0.8553–20.70
h	Normal distribution	One-way ANOVA	−8.720 to −2.726
i	Normal distribution	One-way ANOVA	2.081–8.610
j	Normal distribution	One-way ANOVA	−11.40 to −1.271
k	Normal distribution	One-way ANOVA	−22.71 to −3.676
l	Normal distribution	One-way ANOVA	0.1384–8.060
m	Normal distribution	One-way ANOVA	2.606–7.030
n	Normal distribution	*t* test	−5.397 to −0.7243
o	Normal distribution	Linear Regression	Slope 95% CI: 0.2513–2.790
p	Normal distribution	Linear Regression	Slope 95% CI: 0.3514–1.937
q	Normal distribution	Linear Regression	Slope 95% CI: −0.189 to 0.891
r	Normal distribution	Linear Regression	Slope 95% CI: 0.0744–1.204
s	Normal distribution	Two-way ANOVA	−0.3064 to −0.07172
t	Normal distribution	Two-way ANOVA	−0.1778 to −0.002775
u	Normal distribution	Two-way ANOVA	−0.2946 to −0.01059
v	Normal distribution	Two-way ANOVA	−0.1942 to −0.03028
w	Normal distribution	Two-way ANOVA	−0.3187 to −0.05020
x	Normal distribution	Two-way ANOVA	−0.3454 to −0.04435
y	Normal distribution	Two-way ANOVA	0.002719–0.2159
z	Normal distribution	Two-way ANOVA	0.06831–0.3622
aa	Non-normal	Mann Whitney *U* test	0.04579–1.417
ab	Non-normal	Mann Whitney *U* test	0.06866–0.5624
ac	Normal distribution	*t* test	10,716–8,233,887
ad	Normal distribution	*t* test	478,164–3,712,384
ae	Normal distribution	*t* test	0.6423–9.446
af	Normal distribution	*t* test	−17.07 to −2.215
ag	Normal distribution	*t* test	−14.15 to −4.105
ah	Normal distribution	*t* test	−4,454,906 to −331,789

The Result column indicates superscript letters for each statistical result presented in this section.

## Results

### Maternal and early postnatal outcomes

Maternal mean (±SEM) plasma concentration of MO was 2825.5 ± 35.0 and 521.7 ± 73.6 ng/ml, respectively, at 1 and 4 h after subcutaneous injection. By 20 h, concentrations were below the lower limit of detection of the assay (2 ng/ml; Table 2). These findings are consistent with the rapid plasma clearance of MO and an approximate half-life of 1–2 h. Concentrations of MO in placental tissue showed a similar relationship to that of plasma, with high tissue concentrations at 1 h similarly decreasing by 4 h. Fetal brain tissue concentrations were approximately half those of placental tissue and confirmed placental transfer and transport of MO to the fetal brain. The tissue concentrations at 20 h were not determined.

**Table 2:** Morphine concentrations measured by tandem mass spectrometry

Tissue	Time (h)	Total morphine ± SE
Maternal plasma (*n* = 2)	1	2825.5 ± 35.0 (ng/ml)
Placenta (*n* = 7)	1	2704.6 ± 99.4 (ng/g)
Fetal brain (*n* = 7)	1	1267.2 ± 45.4 (ng/g)
Maternal plasma (*n* = 3)	4	521.7 ± 73.6 (ng/ml)
Placenta (*n* = 11)	4	415.4 ± 10.4 (ng/g)
Fetal brain (*n* = 11)	4	434.0 ± 11.2 (ng/g)
Maternal plasma (*n* = 2)	20	<2 ng/ml

Total morphine detected at 1, 4, and 20 h after maternal injection in maternal plasma. Tissue levels in placenta and fetal brain samples were measured at 1 and 4 h only.

Maternal MO treatment did not affect gestational weight gain or pup birth weight (Extended Data Fig. 1-5*A*,*B*)^a^. There were also no effects on gestational duration, litter size, or maternal nest quality/size (Extended Data Fig. 1-6). Acutely, MO caused observable increases in locomotion. In quantifying the average time spent in the nest during 2 min bins in the first postnatal week,^b^ there was a significant drug × time interaction (*F*_(3,30)_ = 6.1; *p* = 0.0022] and main effects of drug (*F*_(1,10)_ = 11.8; *p* = 0.0063) and time (*F*_(3,30)_ = 4.6; *p* = 0.0087; Extended Data Fig. 1-5*C*). At BL immediately before and immediately after injection (0 h), MO-treated and SAL-treated dams spent comparable amounts of time in the nest. At 1 h postinjection, MO-treated dams spent significantly less time in the nest (*p* < 0.0001) but recovered by 4 h postinjection. MO dams returned their pups to the nest faster on average than SAL dams in the pup retrieval test conducted at BL (*t*_(13)_ = 2.5; *p* = 0.028^c^; Extended Data Fig. 1-5*D*). At weaning, there were no effects of drug on offspring body weight (Extended Data Fig. 1-7). At P42, there was a main effect of sex, with females weighing less than males, but there remained no effect because of drug (Extended Data Fig. 1-7).

### Social behavior and palatable food/sucrose preference testing

In the three-chambered social interaction test at 5–6 weeks of age, there was a main effect of drug in the social preference portion. All animals demonstrated a social preference (*p* < 0.05 vs 50%); however, MO offspring displayed a significant increase in preference for the mouse versus empty cup compared with SAL offspring^d^ (*F*_(1,40)_ = 5.8; *p* = 0.02; Fig. 2*A*). There was no effect of drug or sex and no sex × drug interaction in the social novelty portion of the test (Fig. 2*B*). At 8–9 weeks of age, there were no group differences in consumption or preference of either 60% high-fat diet or 4% sucrose solution (Extended Data Fig. 2-1).

10.1523/ENEURO.0238-22.2022.f2-1 Figure 2-1 High-fat and sucrose preference tests. ***A***, ***B***, There were no group differences in high-fat (***A***) or sucrose preference (***B***) tests. Download Figure 2-1, TIF file.

### Adult operant behavioral testing

In FR1, animals learn that each correct touch results in a single reward, which is the basic behavior required for subsequent operant tasks. All female mice acquired the FR1 criterion (≥70 trials in 30 min) in 7 d, while the males took 11 d. In FR1, male MO offspring learned FR1 significantly slower compared with male SAL offspring (χ^2^ = 4.2; *p* = 0.04^e^; Fig. 2*C*), while females did not differ in FR1 acquisition (Fig. 2*D*). In PR, where the number of touches required for a single reward steadily increases, male MO offspring had a decreased break point (*t*_(20)_ = 3.3; *p* = 0.0033^f^; Fig. 2*E*), while break point did not differ in the females (Fig. 2*F*).

In the 5CSRTT, mice begin by learning the task with a 16 s stimulus, where they encounter a finite stimulus presentation for the first time. All mice took a minimum of 3–5 d of training on the 16 s stimulus before any mouse reached criterion (3 d for females, 5 d for males). Across the first 5 d of the 16 s stimulus training in males, there were main effects of drug on accuracy, omissions, and premature responses (*F*_(1,20)_ = 5.1; *p* = 0.035^g^; *F*_(1,20)_ = 15.9; *p* = 0.0007^h^; *F*_(1,20)_ = 11.7; *p* = 0.0027^i^). Male MO offspring had decreased accuracy, an increased percentage of omissions, and a decreased number of premature responses without a change in the total number of trials achieved (Fig. 3*A–D*). In females, analysis across the first 3 d of the 16 s stimulus training revealed no change in accuracy or omissions (Fig. 3*E*,*F*) but significant main effects of drug on premature responses (*F*_(1,20)_ = 6.8; *p* = 0.017^j^) and total number of trials (*F*_(1,20)_ = 8.4; *p* = 0.009^k^; Fig. 3*G*,*H*), such that female MO offspring made more premature responses and also achieved a greater number of total trials.

We then analyzed the first day that each mouse achieved criterion (e.g., had demonstrated mastery of the task) for each of the stimulus lengths. This analysis revealed the main effects of drug in the males on accuracy (*F*_(1,18)_ = 4.7; *p* = 0.043^l^) and premature responses (*F*_(1,18)_ = 20.9; *p* = 0.0002^m^) . Male MO offspring were less accurate, had a comparable percentage of omissions, but again had decreased premature responses (Fig. 3*I–K*). There were no differences in the females and no differences in trials achieved in either sex. On the final day of testing with a titrated stimulus, male MO offspring made fewer premature responses (*t*_(18)_ = 2.7; *p* = 0.013^n^; Fig. 3*L*). However, there were no differences in either sex for any other error type, total trials, and minimum or median stimulus achieved in titration.

### Behavioral correlations

Behavioral testing revealed that male MO offspring had increased social preference, decreased learning in FR1, decreased motivation in PR, reduced 5CSRTT accuracy, and increased omissions with reduced premature responses. Female MO offspring were less affected, only displaying increased social preference and premature responses. To determine whether any of these behavioral responses were related, correlation analyses were conducted across behavioral outcomes (Fig. 4*A*,*B*). In both males and females, we found a positive correlation between premature responses and correct responses during the training phase of the 16 s 5CSRTT stimulus (males: *r* = 0.73, *p* = 0.0002; females: *r* = 0.64, *p* = 0.0012; Fig. 4*A–D*). In males, this positive correlation was present in both the SAL group (*r* = 0.67, *p* = 0.024^o^) and the MO group (*r* = 0.79, *p* = 0.011^p^; Fig. 4*C*). In females, this correlation between premature and correct responses was only observed in SAL offspring, but did not reach significance in MO offspring that had increased premature responses (female MO: *r* = 0.42, *p* > 0.05^q^; female SAL: *r* = 0.68, *p* = 0.03^r^; Fig. 4*D*). Despite premature responses typically indicating increased impulsivity, this suggests that a tonic level of premature responding may be a learning strategy that is lacking in male MO offspring. Female MO offspring, however, displayed increased premature responding that did not correlate with an increase in correct responses.

### P21 gene expression

Analysis of differential gene expression in the mPFC via qPCR identified significant main effects of drug for six genes, with MO-exposed offspring having increased expression of integrin alpha M (ITGAM; *F*_(1,20)_ = 11.3; *p* = 0.0031^s^), MYD88 (*F*_(1,20)_ = 4.6; *p* = 0.044^t^), DLG4 (*F*_(1,20)_ = 5.02; *p* = 0.037^u^), catechol-O-methyltransferase (COMT; *F*_(1,20)_ = 8.16; *p* = 0.0098^v^), PNOC (*F*_(1,20)_ = 8.2; *p* = 0.0095^w^), and DNMT3A (*F*_(1,20)_ = 7.3; *p* = 0.014^x^; Fig. 5*A–F*). In the AMG, there was one significant main effect of drug: SLC17A7 expression was decreased in MO offspring (*F*_(1,19)_ = 4.6; *p* = 0.045^y^; Fig. 5*G*). In the VTA, TLR4 displayed a significant drug × sex interaction (*F*_(1,18)_ = 9.5; *p* = 0.0065^z^), with male MO offspring having reduced TLR4 expression and no difference in female offspring (Fig. 5*H*). There were no other effects of drug or other drug × sex interactions.

10.1523/ENEURO.0238-22.2022.f5-1 Figure 5-1 P21 fold change values by group (mean ± SEM) for observed sex differences in gene expression. Download Figure 5-1, DOCX file.

There were significant sex effects in the AMG with females having increased expression of DLG4, DNMT1, ITGAM, MBP, and SETD7. Females also had decreased AMG OPRK1 expression compared with males. In the NAc, females had increased expression of MYD88 and TLR2 (Extended Data Fig. 5-1).

### Adult gene expression

In the AMG of adult offspring that had completed operant testing, male MO offspring had increased expression of IL-1β (*U*_(1,20)_ = 28; *p* = 0.033^aa^) and neurotensin (NTS; *U*_(1,20)_ = 21; *p* = 0.0083^ab^) compared with male SAL offspring (Fig. 5*I*,*J*). There were no significant effects of drug on gene expression in the adult female AMG or adult mPFC in either sex.

### Adult immunohistochemistry

Images were captured in the m-mPFC, c-mPFC, MeA (Fig. 6*A–C*), and BLA. In the m-mPFC, analysis of 20× image magnification showed significant increases in both Iba1 and CD68 integrated density normalized to the cell count in female MO versus SAL offspring (*t*_(6)_ = 2.45; *p* = 0.0496^ac^; *t*_(6)_ = 3.2; *p* = 0.019^ad^; Fig. 6*D*,*E*), with no difference in male animals (Extended Data Fig. 6-1). At 40× in the m-mPFC, female MO offspring had significantly increased Iba1 integrated density within the Iba1 cell soma (*t*_(3)_ = 3.65; *p* = 0.036^ae^; Fig. 6*G*, Extended Data Fig. 6-2*A*,*B*). Male MO offspring, however, had reduced Iba1 integrated density within the Iba1 cell soma in the m-mPFC (*t*_(8)_ = 2.99; *p* = 0.017^af^; Fig. 6*H*, Extended Data Fig. 6-2*C*,*D*) and reduced CD68 integrated density within the Iba1 cell soma in the c-mPFC (*t*_(7)_ = 4.3; *p* = 0.0036^ag^; Fig. 6*I*, Extended Data Fig. 6-2*E*,*F*). This was observed at 40×, and no male differences were observed in the mPFC in the 20× images.

10.1523/ENEURO.0238-22.2022.f6-1 Figure 6-1 Male m-mPFC values for Iba1 and CD68 reported as the mean ± SEM. Download Figure 6-1, DOCX file.

10.1523/ENEURO.0238-22.2022.f6-2 Figure 6-2 Representative images for CD68/Iba1 immunohistochemistry. ***A–D***, Representative m-mPFC Iba1/CD68 40× images of female SAL (***A***) and female MO (***B***) depict increased Iba1 in female MO offspring; male SAL (***C***) and male MO (***D***) depict reduced Iba1 in male MO offspring. Representative c-mPFC CD68 40× images of male SAL (L) and male MO (O) depict reduced CD68 in male MO offspring. Download Figure 6-2, TIF file.

In the MeA, 20× analysis revealed that male MO offspring had significantly reduced CD68 integrated density normalized to the cell count (*t*_(8)_ = 2.7; *p* = 0.028^ah^; Fig. 6*C*,*F*). This effect was not present in the BLA or in the females, and no effects were observed looking at individual cells at 40×.

### P21 mPFC RNA-seq

RNA-seq on P21 samples from the mPFC was conducted as a hypothesis-generating experiment, creating gene lists in both male and female offspring of differential gene expression (MO vs SAL: unadjusted *p* < 0.05 and log2FoldChange > 1.2 or less than −1.2). In male offspring, this produced 18 genes that were upregulated and 15 genes that were downregulated in MO compared with SAL (Fig. 7*A*). In female offspring, this produced 31 genes that were upregulated and 11 genes were downregulated in MO compared with SAL (Fig. 7*B*). In the males, gene set enrichment analysis indicated that upregulated genes had significant representation of pathways involving vasopressin (AVP)-like receptors/synthesis and opioid/prodynorphin, along with ontologies involved in histone methylation, DNA methylation, and DNA alkylation (*p*_adjusted_ < 0.05). This was mediated by upregulation of oxytocin (OXT), AVP, DNMT3B, DNMT3L, and MYB in male MO offspring (highlighted in Fig. 7*A*). Downregulated male genes did not produce any significantly enriched pathways with relevance to the CNS. Upregulated genes in female MO offspring aligned significantly with pathways involving toll-like receptor signaling and IL-17 signaling, along with ontologies related to the cytoskeleton, extracellular matrix, endocytic vesicles, and secretory granules (*p*_adjusted_ < 0.05). This was driven by the upregulation of genes S100A7A, S100A8, S100A9, KRT17, DSP (desmoplakin), HPX (hemopexin), FGL1, FGB (fibrinogen beta chain), and LTF (lactoferrin) in female MO offspring (highlighted in Fig. 7*B*). Downregulated female genes had significant enrichment of complement activation, mediated by downregulation of C7 (Fig. 7*B*).

In both males and females, unsupervised gene × drug clustering with the Spearman rank similarity measure correctly clustered the MO versus SAL groups, using either hierarchical or k-means. Genes also correctly clustered in their respective upregulated versus downregulated groups. The Pearson similarity measure did not accurately cluster male or female MO versus SAL groups, using either hierarchical or k-means approaches. In examining the heatmaps, this could be because of high animal–animal variability in gene expression within groups that is better suited for the nonparametric Spearman rank-based approach instead of the Pearson mean-based approach.

## Discussion

This study represents an important advancement in the field by demonstrating (1) construct validity for a mouse model of perinatal morphine exposure that results in executive function deficits in males and (2) evidence in support of alterations in neuron–microglial signaling that may underlie these behavioral deficits. We found that male MO offspring had delayed learning in FR1 and reduced motivation in PR, along with impaired accuracy and decreased attention in the 5CSRTT executive function task. At the end of maternal MO exposure on P21, we detected an increase in gene expression in the microglial pruning gene ITGAM (CR3/CD11b) and neuronal postsynaptic marker DLG4 [postsynaptic density-95 (PSD-95)] in the mPFC in both sexes. Simultaneously, there was a reduction in presynaptic glutamatergic marker SLC17A7 (VGluT1) in the AMG of MO offspring at the same age. In adulthood, female MO offspring had increased Iba1 and CD68 immunolabeling in the mPFC, while these markers were reduced in the mPFC and AMG of male MO offspring.

We treated dams with MO daily for 7 weeks to model long-term maternal opioid use and to include all stages of early neurodevelopment. As a result, female MO offspring had increased impulsive errors during the initial learning of the easiest version of the task with the longest stimulus length. However, this effect was absent once females had successfully learned the task. In contrast, male MO offspring showed increased omissions (indicating inattention), and decreased accuracy in the early stage of learning, with reduced accuracy persisting even after successfully learning the task at progressively shorter stimulus lengths. Further, male MO offspring had reduced premature/impulsive errors throughout. The reduction in impulsivity in male MO offspring is reminiscent of reduced impulsivity in a rat model of early life stress (Sanchez et al., 2021). While reductions in impulsivity are typically seen as beneficial, male MO offspring also had significantly increased inattentive/omission errors during the early stages of learning the longest stimulus duration in the 5CSRTT. Children exposed to opioids prenatally are more likely to exhibit attention deficit hyperactivity disorder symptoms and diagnoses (Haugland et al., 2018; Azuine et al., 2019). Impulsivity and inattention are categorized together as problems with self-regulation (Nigg, 2016) and are highly correlated in naturalistic settings, such as in caregiver reports for children with attention deficit hyperactivity disorder (Bezdjian et al., 2009). However, naturalistic settings allow these behavioral characteristics to manifest across a broad range of activities, with inattention emerging as poor allocation of cognitive processes and impulsivity emerging as poor control over motor or emotional responses (Nigg, 2016). Laboratory tests are limited to a singular activity, with the 5CSRTT measuring impulse control and attention via response variations of the same trial (either before or after the stimulus). Therefore, if male MO offspring are likely to omit responding altogether, it is unlikely they will make many anticipatory responses before stimulus presentation. Importantly, we found a strong positive correlation between impulsive errors and correct responses, demonstrating that some level of impulsive responding may actually facilitate performance in the 5CSRTT, a response pattern that is absent in male MO offspring. Future work should include different executive function tasks to further contextualize these findings, as impulsivity can manifest in a variety of ways (Robinson et al., 2009). Finally, the observed inattention and reduction in impulsivity in males contrasts with previous work reported in a rat model of prenatal MO exposure in which male MO offspring had increased impulsivity in the shortest stimulus duration of the 5CSRTT (Alaee et al., 2021). This previous study used MO exposure only during late gestation, suggesting that executive function outcomes vary based on the timing and/or duration of opioid exposure during pregnancy.

We assessed social behavior because PFC–AMG circuitry is important for social preference (Huang et al., 2020) and PFC–AMG functional connections are particularly vulnerable to the effects of prenatal opioid exposure (Radhakrishnan et al., 2021). We found that in adolescence, male and female MO offspring had increased social preference but no change in social recognition in the three-chambered social interaction test. This is consistent with a study showing that male offspring exposed to MO during middle and late gestation had increased social play and social approach behavior (Hol et al., 1996). Unlike executive function, opioid effects on social behavior may be less dependent on the timing of exposure during pregnancy and less variable based on sex. In conjunction with this increase in social preference in MO offspring, we saw an increase in PSD-95 expression in the mPFC and reduced VGluT1 expression in the AMG and at P21, emphasizing the vulnerability of the mPFC and AMG to the effects of opioids perinatally, particularly as it relates to excitatory neurotransmission signatures. Loss of PSD-95 reduces sociability in adolescence and increases PFC NMDA/AMPA receptor tone (Coley and Gao, 2019), so increases in PFC PSD-95 may contribute to the increased sociability we observed. Social encounters are also rewarding, so this may reflect increased reward-seeking behavior in MO offspring, which is supported by an increase in NTS expression in the AMG of adult MO offspring. Neurotensin in the central AMG has been shown to be reinforcing (Torruella-Suárez and McElligott, 2020). This is also consistent with reports of enhanced drug self-administration in MO offspring (Ramsey et al., 1993; Hol et al., 1996; Grecco and Atwood, 2020); however, this may depend on reward modality as we did not find an increase in reward-driven behavior measured by high-fat diet and sucrose preference tests.

We saw an increase in ITGAM (CR3) and MYD88 expression in the mPFC at P21 in MO offspring, which is consistent with the ability of MO to activate the TLR4 pathway (Hutchinson et al., 2010; Wang et al., 2012) and a report of late gestational methadone exposure inducing a signature of brain TLR4 activation in offspring at P10 (Jantzie et al., 2020). RNA-seq and gene enrichment analysis at P21 revealed several pathways that were significantly altered by perinatal MO exposure and were mutually exclusive based on sex. Female MO offspring had upregulated genes with significant enrichment in pathways related to immune signaling, while males had enrichment in pathways related to OXT/AVP and DNA methylation. Male MO offspring had a reduction in adulthood CD68 protein in the mPFC and medial AMG, indicating a possible lasting impairment in microglial phagocytosis. This idea is supported by emerging evidence that microglial phagocytosis was indeed impaired in the nucleus accumbens of male offspring exposed prenatally to oxycodone (Smith et al., 2022b), so we suggest this may also be the case in the male mPFC and AMG. In the nucleus accumbens, male oxycodone-exposed offspring had reduced dopamine D_1_ receptor microglial engulfment during both adolescence and adulthood. This is relevant to our work because D_1_ receptor pruning by microglia in males drives the developmental decrease in social play (Kopec et al., 2018), indicating that perinatal opioid exposure may impair microglial-mediated pruning and prevent a normal developmental reduction in social play that we captured via increased social preference. It was interesting that AMG reductions in CD68 protein in adult male MO offspring coincided with an increase in IL-1β gene expression, which may suggest that IL-1β dysregulation and changes in microglial phagocytosis co-occur in the AMG of male MO mice. We acknowledge the limitations of small sample sizes for some groups in the immunohistochemistry analysis and the exploratory nature of our RNA-seq analysis. Nonetheless, the gene expression data combined with histologic examination provide consistent evidence of the importance of alterations in neuron–microglia signaling. Future work will need to examine sex-specific developmental trajectories of microglial involvement in PFC and AMG pruning, along with how perinatal opioid exposure may disrupt these processes and the relationship to executive function and social behavior. This will likely include circuit-level dissection of prelimbic versus infralimbic mPFC and medial versus basolateral AMG contributions to the pattern of behavioral changes we observed. We acknowledge that a limitation of our current study is the lack of differentiation between the prelimbic and infralimbic mPFC, which should be addressed in subsequent studies. We also plan to prioritize P21 histologic assessment in future work, along with other early postnatal time points that are relevant to windows of synaptic pruning.

In addition to the PFC and AMG being affected by perinatal MO, we found a reduction in TLR4 expression in the male VTA at P21. TLR4 signaling in the VTA is important for morphine conditioned place preference (Chen et al., 2017), which is enhanced in offspring exposed to MO in late gestation (Gagin et al., 1997). Conditioned place preference is a behavioral assay that relates to reward-driven behavior and motivational state (McKendrick and Graziane, 2020), so the decreased TLR4 expression could possibly relate to reduced motivation observed in males. While more work is needed, it is possible that the reduction in adult male microglial markers of motility and phagocytosis may underlie the constellation of behavioral changes observed in males, while the increase in females could help prevent many (but not all) of these behavioral changes. Results from the RNA-seq analysis of P21 mPFC provide additional insight. Instead of TLR4 induction, male MO offspring had an upregulation of OXT and AVP. OXT and AVP signaling in the PFC are both important for motivated social behavior (Caldwell and Albers, 2016). During human development from infancy to childhood, peripheral OXT and attention to social cues decrease with age, while attention to nonsocial cues is enhanced (Nishizato et al., 2017). Although speculative and requiring further work, our data may suggest the possibility that male MO offspring could maintain higher OXT levels and enhanced social preference at the cost of nonsocial attention assessed in the 5CSRTT.

Finally, we detected increased COMT and PNOC expression in the P21 mPFC of MO exposed offspring, which is consistent with clinical literature that has identified single nucleotide polymorphisms in COMT and PNOC as driving individual differences in NOWS outcomes (Wachman et al., 2013, 2015, 2017). The PNOC gene encodes prepronociceptin and is involved in both opioid signaling and processing motivationally relevant stimuli (Rodriguez-Romaguera et al., 2020). The COMT gene encodes catechol-O-methyltransferase, an enzyme that degrades catecholamines and specifically degrades at least 50% of the dopamine in the PFC (Käenmäki et al., 2010), making it important for executive function (Shen et al., 2014). Genotypic variations that increase COMT activity were associated with longer NOWS recovery time in infants and poorer executive function performance in adults (Shen et al., 2014; Wachman et al., 2017). Increases in mPFC COMT expression could relate to executive function deficits observed in the 5CSRTT. This may be important for clinically individualized approaches because a genotypic COMT variation could identify an increased risk of executive function deficits after prenatal opioid exposure. We also saw increased gene expression of DNMT3A in the P21 mPFC, which is important for *de novo* DNA methylation. RNA-seq in male MO offspring revealed DNA methylation as a significantly enriched upregulated pathway, mediated by DNMT3B, DNMT3L, and MYB expression. DNA methylation patterns are highly responsive to environmental challenges during the prenatal period. Clinically, infants treated for NOWS had hypermethylation of the μ opioid receptor gene (OPRM1), and the degree of hypermethylation corresponded to NOWS severity (Wachman et al., 2014; McLaughlin et al., 2017). Increased expression of genes involved in DNA methylation in male MO offspring suggests that this may also relate to long-term behavioral outcomes, although future studies will need to confirm this functionally and identify targets of possible increased methylation.

The 7 week daily maternal MO injection protocol is a model of opioid use before and during pregnancy, including exposure via lactation to model the third trimester brain development in humans (Livy et al., 2003). The 10 mg/kg dose at 1 and 4 h postinjection produced maternal plasma MO concentrations in mice that were within the same range observed in human mothers testing positive for MO via urinalysis (Smith et al., 2022a) and were undetected at 20 h. MO metabolism produces comparable concentrations in plasma and urine (Lafolie et al., 1996), so the dose used here is likely to be clinically relevant. There were transient changes in maternal behavior 1 h after MO injection that rapidly normalized, so this protocol did not significantly impair maternal behavior. The disruption of the circadian cycle caused by moving dams and offspring to a reversed light cycle on P14 could be a stressor that may affect development. Although the SAL and MO groups experienced this equally, this could be a topic of future investigation.

In conclusion, perinatal MO exposure during pregestation, gestation, and lactation causes sex-dependent behavioral and molecular responses in the brain, with male offspring being behaviorally more affected than females. Executive function outcomes seem to depend on sex and the timing of opioid exposure during gestation, while social preference does not. The heightened social preference and impaired learning, motivation, attention, and accuracy in male MO offspring could relate to lasting impairments in microglial phagocytic capacity. Female MO offspring appear to have evidence of increased microglial phagocytosis, which potentially could dampen a subset of the behavioral effects.

## Synthesis

Reviewing Editor: Erica Glasper, The Ohio State University Decisions are customarily a result of the Reviewing Editor and the peer reviewers coming together and discussing their recommendations until a consensus is reached. When revisions are invited, a fact-based synthesis statement explaining their decision and outlining what is needed to prepare a revision will be listed below. The following reviewer(s) agreed to reveal their identity: Marie-Ève Tremblay.

Manuscript Name: Perinatal morphine exposure leads to sex-dependent executive function deficits and microglial changes in mice

Manuscript #: eN-NWR-0238-22

Synthesis: In this study, the authors utilize a mouse model of perinatal morphine exposure to demonstrate sex-specific deficits in executive function and alterations in neuron-microglial signaling that may underlie these behavioral deficits. This work is timely and of importance because the opioid epidemic continues to be a challenge and our understanding of the long-term consequences to the health of exposed children is still unknown. The authors report that male mice exposed to morphine experienced a delay in learning and possible reduced motivation during the fixed ratio task. Deficits in attention and accuracy of executive functioning were also observed in male mice exposed to neonatal morphine. Neonatal morphine exposure impaired cognitive functioning of female mice, but to a lesser extent than males. Gene expression in the prefrontal cortex (PFC), ventral tegmental area (VTA) and amygdala revealed differences in both adolescence and adulthood. Microglial assessment, via IBA1 specific immunohistochemistry revealed significant differences in adulthood, with females exhibiting more IBA1- positive cells and greater CD68 levels but males demonstrating lower CD68 levels in the medial amygdala. Transcriptional profiling, via RNA-seq, demonstrated sex differences at postnatal day 21 in the PFC of morphine-exposed mice. The findings in this paper are interesting. The manuscript is well written and there is clear scientific rationale. There are methodological details, however, that need to be clarified to allow the reviewers, and reviewing editor, to fully evaluate the suitability of this manuscript for publication. Additionally, the authors are encouraged to include estimation statistics in the revised submission, if appropriate. Specific reviewers’ comments have been directly placed into this synthesis and are italicized.

Major Comments

Introduction

• Page 5 - The authors refer to their model “a 7-week prenatal morphine model” but the exposures are not limited to in utero exposure. It may be more appropriate to use a broader, more temporally-inclusive, term like 7-week early life exposure model?

•

Methods

• Page 6, Experimental design and maternal morphine exposure

o After combining small litter sizes, the final maternal sample size is 7 MO litters and 6 SAL litters. Please provide a table (as an extended data file) that includes the following information: Number of offspring in each group for each endpoint and variable measures

o Please discuss how litter effects were controlled for if multiple offspring from the same litter were evaluated.

o Please justify the use of a small behavioral n size (n=6 mice/group).

o Please provide rationale for moving the dams and litters to a reversed light/dark cycle on P14 and not habituating the dams to a reversed light/dark cycle prior to breeding? Please include discussion of the extent to which disruptions in the circadian cycle may alter development (e.g., multiple hit model).

• Page 7, Lines 130-141 - The numbers of mice used to evaluate morphine exposure during the second week of gestation are very low (n = 2-3). While an n of 2 is appropriate for validating the measurement of morphine, please explain how adequate sample sizes were determined for the other studies, including maternal, fetal, and placental tissue studies.

• Pages 7-10 - Given that the mice remained in a reversed light/dark cycle after weaning, please state the time of day for each behavioral test.

• Page 7, Pup retrieval test - Please answer the following questions

o Was the test performed during the dark phase?

o If so, were red lights used to visualize retrievals on the video recording?

o What measures were evaluated during this test?

• Latency to first pup retrieval?

• Duration to retrieve all pups?

• Number of pups retrieved during the 10 minute test?

• Was this test evaluated by humans or by computer software? If humans, were they blinded to groups and what was the inter-rater reliability? If software, how was the software validated?

• Page 8, Three chambered social interaction test (3C)

o Please discuss how the mice were habituated to the lighting conditions during the 3C social interaction test. Specifically, how could the use of dim white light alter their social behaviors?

o Please describe the main outcome measures of interest, including the following: time spent with novel conspecific, frequency of visits

o Please discuss whether a preference index was calculated.

o Discuss whether this test was evaluated by humans or by computer software? If humans, were they blinded to groups and what was the inter-rater reliability? If software, how was the software validated?

• Page 7, Immunohistochemistry

o Please provide product number for all antibodies used.

o Since there are marked differences in microglia and connectivity in cortical subregions (e.g., infra and prelimbic cortex), the authors should perform their immunohistochemical analyses by sub-region in the mPFC and amygdala.

• Page 11, Gene expression - Please include details related to method of euthanization. Also, were the brains perfused or were non-perfused brains used.

• Page 15, Immunohistochemistry

o The authors state that bilateral images of BLA and MeA were obtained, but only the MeA results were presented.

o Please provide greater clarity regarding how density measurements were obtained.

o Please discuss why immunohistochemistry for IBA1 was only investigated in adult mice. Given the authors finding differences in gene expression at P21, a developmental effect of synaptic pruning of microglia may have been observed.

Results

• Please make sure that readers understand that the superscript letters refer to the 1st column in the Table 1.

• Please consider showing including the negative data in graphs or in additional tables instead of writing “data not shown” within the text.

• Page 17, Statistical table - The presentation of the information withing the statistical table could be improved and its references within the text could be made clear. Listing the comparisons being made within the table could also provide clarity. The authors may also consider adding additional statistical information within the text (e.g., F/t- and p-values).

• Page 20, Adult operant behavioral testing - Please report if overall sex differences in the acquisition of FR or 5CSRTT exist.

• Page 22, Behavioral correlations - Can the following sentence be supported by the data? “Despite premature responses typically indicating increased impulsivity, this suggests that a tonic level of premature responding may be a learning strategy that is lacking in male MO offspring.” If anything, because the female morphine offspring have increased premature responses that do not significantly positively correlate with correct answers, does this not mean that females have increase impulsivity?

Figures

• Please use one term to represent the neonatal morphine model. It is called different names in Figure 1 and 2, as well as in other places.

• Figure 2 - Please determine whether an asterisk is missing in the legend (i.e., next to “MO”).

• Figure 7 - Please consider making the yellow bars on the RNAseq data more transparent so that the gene names can be easily read.

Updates to Existing Language

• The authors should refrain from referring to microglial morphology or function as “microglial activation. A recent consensus paper from leaders in the field may be of use: https://papers.ssrn.com/sol3/papers.cfm?abstract_id=4065080.

• Please consider gender neutral language with regards to humans (e.g., substance testing in pregnant people).

• Please consider updating language around substance use according to these guidelines to reduce stigma https://www.canada.ca/en/public-health/services/publications/healthy-living/communicating-about-substance-use-compassionate-safe-non-stigmatizing-ways-2019.html

• The language focusing on the male changes biases the reader from focusing on the effects of morphine in females (potentially increase impulsivity, increased IBA1, CD68, complement RNA-seq pathways). The authors should be more careful and specific in their language avoiding phrases such as “largely male-specific” and focus on the specific findings in males and females.

Supplemental Figures

• Figure 3 - Since the authors report significant effects of morphine exposure on IBA1 density, the authors must include represented photomicrographs for all groups (sex * rearing).

• Please use the term “Extended Data” instead of “Supplemental” to increase ease of publication, should the manuscript be deemed suitable for publication within eNeuro.

Minor Comments

Title Page

• Please consider changing the title to: Perinatal morphine exposure leads executive function deficits and microglial changes in male mice

Introduction

• Page 3

o Please include the incidence, and not just the percent change, of Neonatal Opioid Withdrawal Syndrome

o Please indicate whether alterations in cognition persist in adulthood and if there are any sex/gender differences as a result of neonatal exposure to opioids.

Methods

• Page 5 - Please clarify why the authors did not use B6D2F1/J hybrid mice dams. While offspring have increased genetic diversity, the dams do not have genetic diversity.

• Page 6

o Please discuss when the small litters were combined.

o Please clarify whether the dams that were gestationally administered morphine, but had their litters combined, were assessed.

• Page 8, Line 167 - Please provide the catalog for the high fat diet chow.

• Page 11, Gene expression - Please provide rationale for examining the VTA and NAc.

• Page 17, Statistics

o Please spell out “RM”. This should read “repeated measures”.

o Please be consistent in the presentation of SEM or SD. Ensure that the correct error variance is consistently described in the results sections.

Figures

• Figure 1 - Please designate GD-7 the start of the study.

• Figure 2

o Please justify separating the data in Figures 2E and F into separate graphs when Figures 2A and B are on the same graph.

o Please make the Y-axes in Figures 2E and 2F the same scale.

• Figure 3 - Please make the Y-axes for Figures 3A through 2H the same scale.

• Figure 5

o Figure 5H

• Please add a figure legend to Figure 5H and add the appropriate significance mark within the legend.

• Please explain if it’s necessary to use the “#” mark beside the title in Figure 5H. Could the single asterisk above the male morphine group be sufficient?

• Figure 6 - Please provide methodological clarity. mPFC typically refers to medial PFC, not mid PFC. Did the authors separate mid PFC from caudal PFC? If this was the case, please include rationale for this choice.

Discussion

• Page 25 - Please clarify whether the males exposed to neonatal morphine exhibited reduced motivation or delayed learning?

• Please discuss whether children exposed to neonatal opioids demonstrate increased incidence of ADHD.

## Author Response

DETAILED RESPONSE TO SYNTHESIS OF REVIEWS:

We are grateful for the opportunity to submit a revised version of our manuscript and are thankful for the comments, which are addressed in point-by-point responses below and in the highlighted text in the revised submission. We would like to add that the RNAseq dataset is uploaded on GEO and will be released publicly pending manuscript acceptance.

The full statement, “Experimental procedures were all conducted in compliance with the National Institutes of Health Guidelines for the Care and Use of Animals and approved by the [Author University] Institutional Animal Care and Use Committee” must be included in the cover letter with the Author’s Institution named.

RESPONSE: This statement is in the cover letter.

---------------------------------------------

Synthesis of Reviews:

Computational Neuroscience Model Code Accessibility Comments for Author (Required):

N/A

Synthesis Statement for Author (Required):

Manuscript Name: Perinatal morphine exposure leads to sex-dependent executive function deficits and microglial changes in mice

Manuscript #: eN-NWR-0238-22

Synthesis: In this study, the authors utilize a mouse model of perinatal morphine exposure to demonstrate sex-specific deficits in executive function and alterations in neuron-microglial signaling that may underlie these behavioral deficits. This work is timely and of importance because the opioid epidemic continues to be a challenge and our understanding of the long-term consequences to the health of exposed children is still unknown. The authors report that male mice exposed to morphine experienced a delay in learning and possible reduced motivation during the fixed ratio task. Deficits in attention and accuracy of executive functioning were also observed in male mice exposed to neonatal morphine. Neonatal morphine exposure impaired cognitive functioning of female mice, but to a lesser extent than males. Gene expression in the prefrontal cortex (PFC), ventral tegmental area (VTA) and amygdala revealed differences in both adolescence and adulthood. Microglial assessment, via IBA1 specific immunohistochemistry revealed significant differences in adulthood, with females exhibiting more IBA1- positive cells and greater CD68 levels but males demonstrating lower CD68 levels in the medial amygdala. Transcriptional profiling, via RNA-seq, demonstrated sex differences at postnatal day 21 in the PFC of morphine-exposed mice. The findings in this paper are interesting. The manuscript is well written and there is clear scientific rationale. There are methodological details, however, that need to be clarified to allow the reviewers, and reviewing editor, to fully evaluate the suitability of this manuscript for publication. Additionally, the authors are encouraged to include estimation statistics in the revised submission, if appropriate. Specific reviewers’ comments have been directly placed into this synthesis and are italicized.

Major Comments

Introduction

• Page 5 - The authors refer to their model “a 7-week prenatal morphine model” but the exposures are not limited to in utero exposure. It may be more appropriate to use a broader, more temporally-inclusive, term like 7-week early life exposure model?

RESPONSE: We have changed this to read “perinatal” morphine model

•

Methods

• Page 6, Experimental design and maternal morphine exposure

o After combining small litter sizes, the final maternal sample size is 7 MO litters and 6 SAL litters. Please provide a table (as an extended data file) that includes the following information: Number of offspring in each group for each endpoint and variable measures

RESPONSE: We have included this in Extended Data Table 1-1. Thank you for this suggestion, as we believe this makes it much easier to interpret sample sizes.

o Please discuss how litter effects were controlled for if multiple offspring from the same litter were evaluated.

RESPONSE: We did not use any more that 2 per litter in any one experimental group and in most cases we only used 1 per litter, so we did not evaluate litter effects.

o Please justify the use of a small behavioral n size (n=6 mice/group).

RESPONSE: We apologize for the confusion and believe that Extended Data Table 1-1 improves clarity. We did not use n = 6 for behavioral testing. We refer the reviewer to the text Page 7 Line 133-135: The second cohort was used for all behavioral testing and adult gene expression and housed in cages of 3-4 unless otherwise noted (n = 10-12/group; n = 1-2/litter/sex).

o Please provide rationale for moving the dams and litters to a reversed light/dark cycle on P14 and not habituating the dams to a reversed light/dark cycle prior to breeding? Please include discussion of the extent to which disruptions in the circadian cycle may alter development (e.g., multiple hit model).

RESPONSE: We agree that this is not ideal and have added the following statements.

Methods (Page 7 Line 130-132): The timing of the move was dictated by housing constraints within the vivarium, which prevented habituation prior to breeding.

Discussion (Page 33 Line 729-731): The disruption of the circadian cycle caused by moving dams and offspring to a reversed light cycle on P14 could be a stressor that may affect development. Although the SAL and MO groups experienced this equally, this could be a topic of future investigation.

• Page 7, Lines 130-141 - The numbers of mice used to evaluate morphine exposure during the second week of gestation are very low (n = 2-3). While an n of 2 is appropriate for validating the measurement of morphine, please explain how adequate sample sizes were determined for the other studies, including maternal, fetal, and placental tissue studies.

RESPONSE: We have clarified that the small sample size used for this quantification was to verify MO transport to maternal plasma and fetal tissue, not for comparative purposes (Page 8 Line 149-150). For fetal brain and placental validation, we used n = 7-11 samples (Table 2).

• Pages 7-10 - Given that the mice remained in a reversed light/dark cycle after weaning, please state the time of day for each behavioral test.

RESPONSE: We have added that behavioral testing occurred 1 - 8 h after lights off, since the testing took multiple hours. Order was balanced between groups.

• Page 7, Pup retrieval test - Please answer the following questions

o Was the test performed during the dark phase?

RESPONSE: We have clarified that this was performed during the light phase.

o If so, were red lights used to visualize retrievals on the video recording?

N/A

o What measures were evaluated during this test?

• Latency to first pup retrieval?

• Duration to retrieve all pups?

• Number of pups retrieved during the 10 minute test?

• Was this test evaluated by humans or by computer software? If humans, were they blinded to groups and what was the inter-rater reliability? If software, how was the software validated?

RESPONSE: Thank you for pointing out that these details were lacking, we have added them (Page 8 Line 157-160). For pup retrieval and 3-chambered social interaction, a single human investigator evaluated the videos with the assistance of BORIS (Behavioral Observation Research Interactive Software). The advantage of BORIS is that investigators can play video footage frame-by-frame and annotate videos directly, allowing editing of evaluations if there are any questions on the behaviors. This allows us to re-evaluate their annotations at any point, but also make the evaluation process very time consuming. For these reasons, we do not use a second investigator.

• Page 8, Three chambered social interaction test (3C)

o Please discuss how the mice were habituated to the lighting conditions during the 3C social interaction test. Specifically, how could the use of dim white light alter their social behaviors?

RESPONSE: Mice were habituated to the dim white light for 5 min prior to testing (habituation phase). We have added that this was dim “indirect” light to minimize the disturbance. Additionally, order was balanced between groups and all groups were exposed to the same lighting conditions.

o Please describe the main outcome measures of interest, including the following: time spent with novel conspecific, frequency of visits

o Please discuss whether a preference index was calculated.

o Discuss whether this test was evaluated by humans or by computer software? If humans, were they blinded to groups and what was the inter-rater reliability? If software, how was the software validated?

RESPONSE: Thank you again for pointing out that these details were lacking, we have added them (Page 9 Line 176-180).

• Page 7, Immunohistochemistry

o Please provide product number for all antibodies used.

RESPONSE: Product numbers have been added for CD68 and Iba-1 antibodies.

o Since there are marked differences in microglia and connectivity in cortical subregions (e.g., infra and prelimbic cortex), the authors should perform their immunohistochemical analyses by sub-region in the mPFC and amygdala.

RESPONSE: We would like to highlight that we did investigate amygdalar sub regions by analyzing the MeA and BLA separately. We agree that there are important differences in connectivity and function between the infralimbic and prelimbic mPFC. We would like to emphasize that we mention in the discussion that future work needs to include circuit level dissection of the PL & IL mPFC. However, our rationale for imaging the junction between the IL & PL and not analyzing them separately was based on a previous study that investigated Iba1 expression after chronic stress and found no differences between the IL & PL mPFC (Kopp et al., 2013). There were cortical layer differences (layers 2/3 vs. 5/6) but this was in rats where it is easier to clearly dissociate the layers during imaging (Kopp et al., 2013). The focus of our current manuscript is cognitive function and we also found differences in motivation in the males. Due to the rostral-caudal differences in the mPFC as it relates to motivated behaviors and cognitive control (Kouneiher et al., 2009), we chose to image mid vs caudal mPFC to examine any potential rostral-caudal differences. We have added this rationale to the methods section (Page 16 Lines 343-346).

• Page 11, Gene expression - Please include details related to method of euthanization. Also, were the brains perfused or were non-perfused brains used.

RESPONSE: We have added these details “via rapid decapitation without perfusion” and added that brains “were promptly removed and immediately” stored in RNAlater®.

• Page 15, Immunohistochemistry

o The authors state that bilateral images of BLA and MeA were obtained, but only the MeA results were presented.

RESPONSE: We indicate in the results section that there were no effects in the BLA (Page 24 Line 534-535). We also added in the results section that images were obtained in the BLA (Page 24 Line 520).

o Please provide greater clarity regarding how density measurements were obtained.

RESPONSE: We have added that these density measurements are automatic measurement functions in ImageJ.

o Please discuss why immunohistochemistry for IBA1 was only investigated in adult mice. Given the authors finding differences in gene expression at P21, a developmental effect of synaptic pruning of microglia may have been observed.

RESPONSE: This is a great observation and agree that immunohistochemistry at P21 is an important future direction. Now that we have analyzed this dataset from P21, we have added in the discussion that “we also plan to prioritize P21 histological assessment in future work, along with other early postnatal time points that are relevant to windows of synaptic pruning.” (Page 30 Line 672-673) We would like to collect tissue at multiple early postnatal time points for this type of analysis, in order to capture the peak in dendritic spine density and subsequent pruning period for each brain region. This requires a larger sample size than we had available at the time of this initial study.

Results

• Please make sure that readers understand that the superscript letters refer to the 1st column in the Table 1.

RESPONSE: Thank you for catching this, we have made this more clear.

• Please consider showing including the negative data in graphs or in additional tables instead of writing “data not shown” within the text.

RESPONSE: For all data mentioned but “not shown", we have presented results in Extended Data Tables 1-5, 1-6, and 6-1 in the revised version.

• Page 17, Statistical table - The presentation of the information withing the statistical table could be improved and its references within the text could be made clear. Listing the comparisons being made within the table could also provide clarity. The authors may also consider adding additional statistical information within the text (e.g., F/t- and p-values).

RESPONSE: We apologize that this was not clear before. We have added a header to the first column of Table 1 that was missing, which is now entitled “Results.” We have also added a legend describing “The result column indicates superscript letters for each statistical result presented in this section.” We refer the reviewer to the F/t statements with p-values in the results section for each of the statistically significant findings, along with mention of whether this was a main effect or interaction.

• Page 20, Adult operant behavioral testing - Please report if overall sex differences in the acquisition of FR or 5CSRTT exist.

RESPONSE: Yes, we observe such pronounced sex differences that it makes statistical comparisons challenging because data collection ends up being on different days in males vs. females. We have added that in FR1, “All female mice acquired FR1 criterion (70 or more trials in 30 min) in 7 days while the males took 11 days (Page 20 Line 444-446).” This difference prevents direct sex comparisons because all females progressed through training faster than the males so the rest of the testing was conducted on different days in males vs. females. We avoid overtraining females on FR1 to align with the training days in males because we are concerned that this may lead them to develop persistent habitual responding. In the 5CSRTT, females began progressing through the first stimulus in 3 days whereas this takes 5 days in the males (Page 21 Line 453-454).

• Page 22, Behavioral correlations - Can the following sentence be supported by the data? “Despite premature responses typically indicating increased impulsivity, this suggests that a tonic level of premature responding may be a learning strategy that is lacking in male MO offspring.” If anything, because the female morphine offspring have increased premature responses that do not significantly positively correlate with correct answers, does this not mean that females have increase impulsivity?

RESPONSE: We apologize for the confusion and are happy to explain this thought process further. We believe the strong correlation between premature responses and correct responses in male SAL offspring indicate that a degree of premature responding assists the mice in making correct responses (“tonic level of premature responding may be a learning strategy”). Then the male MO offspring displayed reduced premature responses and their premature responses did not correlate with correct responses (“this learning strategy may be lacking in male MO offspring”). We agree with your point on the females and have added “Female MO offspring, however, displayed increased premature responding that did not correlate with an increase in correct responses.” (Page 22-23 Line 491-493)

Figures

• Please use one term to represent the neonatal morphine model. It is called different names in Figure 1 and 2, as well as in other places.

RESPONSE: We understand the desire for consistency and have changed the term to “perinatal” in the introduction, where we also introduce that the perinatal model is used to study prenatal exposure in mice “to include exposure that is equivalent to the third trimester brain development in humans” (Page 5 Line 92-93). From this point forward, we refer to our model as perinatal exposure. We only use the term “prenatal” when referring to exclusive prenatal exposure such as with clinical references or references to exclusive prenatal models.

• Figure 2 - Please determine whether an asterisk is missing in the legend (i.e., next to “MO”).

RESPONSE: We have edited the legend to read MO*.

• Figure 7 - Please consider making the yellow bars on the RNAseq data more transparent so that the gene names can be easily read.

RESPONSE: We apologize for the poor clarity, we have made the gene name text easier to read in the revised version of Figure 7.

Updates to Existing Language

• The authors should refrain from referring to microglial morphology or function as “microglial activation. A recent consensus paper from leaders in the field may be of use: https://papers.ssrn.com/sol3/papers.cfm?abstract_id=4065080.

RESPONSE: Thank you for this reference, although access was restricted for us at the moment. We have removed the term “activation” and instead replaced it with terms that annotate specific function, such as: microglial-mediated pruning, motility, and phagocytosis. (Page 4 Line 77; Page 31 Line 682)

• Please consider gender neutral language with regards to humans (e.g., substance testing in pregnant people).

RESPONSE: We maintain the use of “woman” and “mother” as it refers to previously published clinical studies that used those terms to define research inclusion criteria. Hence, these terms offer the most accurate representation of the existing clinical data. We appreciate the more recent movement to use pregnant people as a gender-neutral term and as clinical research updates the language for study criterion, we can update how we describe these studies in the future. Additionally, we refrain from using gender-based terms in the mouse model and refer only to male vs. female.

• Please consider updating language around substance use according to these guidelines to reduce stigma https://www.canada.ca/en/public-health/services/publications/healthy-living/communicating-about-substance-use-compassionate-safe-non-stigmatizing-ways-2019.html

RESPONSE: We agree with the importance of adhering to these guidelines and thank you for drawing attention to this. We have changed Page 3 Line 54 from polysubstance abuse to “use.” We have also reworded the paragraph on Page 4 Line 61-63 to read “Opioid maintenance therapy with methadone or buprenorphine is recommended for pregnant women who disclose an opioid use disorder, but major barriers can prevent access to these therapies. Hence, morphine remains among the most prevalent opioid detected...”

• The language focusing on the male changes biases the reader from focusing on the effects of morphine in females (potentially increase impulsivity, increased IBA1, CD68, complement RNA-seq pathways). The authors should be more careful and specific in their language avoiding phrases such as “largely male-specific” and focus on the specific findings in males and females.

RESPONSE: In the significance statement we have removed the term “largely male-specific” and removed the summary of the overall lack of cognitive effects in females, now stating that they instead had an increase in impulsivity.

Supplemental Figures

• Figure 3 - Since the authors report significant effects of morphine exposure on IBA1 density, the authors must include represented photomicrographs for all groups (sex * rearing).

RESPONSE: We apologize for the typo in Extended Data Figure 3 F. This is now Extended Data Figure 6-1. We have corrected it to read Male MO c-mPFC instead of mPFC. In the Extended Data Figure A - D, we have included representative merged m-mPFC Iba1/CD68 images for all groups (female SAL, female MO, male SAL, male MO) to demonstrate findings in Figure 6 G & H. For the males, we also show the caudal mPFC (c-mPFC) images on only the CD68 channel to demonstrate the difference found in Figure 6 I. This difference was not observed in the females. We hope this is now more clear after addressing the typo.

• Please use the term “Extended Data” instead of “Supplemental” to increase ease of publication, should the manuscript be deemed suitable for publication within eNeuro.

RESPONSE: We have made this change.

Minor Comments

Title Page

• Please consider changing the title to: Perinatal morphine exposure leads executive function deficits and microglial changes in male mice

RESPONSE: Thank you for this suggestion but we believe that this change would overlook the effects that we do observe in the females (consistent with the previous comment to avoid male bias).

Introduction

• Page 3

o Please include the incidence, and not just the percent change, of Neonatal Opioid Withdrawal Syndrome

RESPONSE: We have added the following sentence: “As of 2017, NOWS rates were estimated at 7.3 per 1000 births, with some states seeing as high as 53.5 cases per 1000 births (Hirai et al., 2021).” (Page 3 Line 37-38)

o Please indicate whether alterations in cognition persist in adulthood and if there are any sex/gender differences as a result of neonatal exposure to opioids.

RESPONSE: We did our best to address this in our original submission via the following sentence: These cognitive problems persist into adolescence (Nygaard et al., 2017) and males appear more vulnerable than females (Hickey et al., 1995; Nygaard et al., 2015). To our knowledge, there are not studies that extend to adulthood and the studies that do exist pre-adulthood are often underpowered to detect sex differences.

Methods

• Page 5 - Please clarify why the authors did not use B6D2F1/J hybrid mice dams. While offspring have increased genetic diversity, the dams do not have genetic diversity.

RESPONSE: All behavioral work within the lab has historically been conducted on B6D2F1/J mice derived from B6 dams, which is done to increase genetic diversity in the offspring. Therefore, this strategy was maintained in the current experiments.

• Page 6

o Please discuss when the small litters were combined.

RESPONSE: We have added that this was conducted on P2 after the pup retrieval test was completed. (Page 6, Line 126)

o Please clarify whether the dams that were gestationally administered morphine, but had their litters combined, were assessed.

RESPONSE: We have added that the dams that no longer had pups were removed from the study on P2. (Page 7 Line 128)

• Page 8, Line 167 - Please provide the catalog for the high fat diet chow.

RESPONSE: Added.

• Page 11, Gene expression - Please provide rationale for examining the VTA and NAc.

RESPONSE: We have added that “We included the NAC and VTA based on reports of increased reward-driven behavior in MO offspring (Grecco and Atwood, 2020; Hol et al., 1996; Ramsey et al., 1993).”

• Page 17, Statistics

o Please spell out “RM”. This should read “repeated measures”.

RESPONSE: We have made this change.

o Please be consistent in the presentation of SEM or SD. Ensure that the correct error variance is consistently described in the results sections.

RESPONSE: We apologize for the inconsistency and have changed Table 2 to report SEM instead of SD.

Figures

• Figure 1 - Please designate GD-7 the start of the study.

RESPONSE: We have added this to the legend for Figure 1.

• Figure 2

o Please justify separating the data in Figures 2E and F into separate graphs when Figures 2A and B are on the same graph.

RESPONSE: Figures 2 A & B were analyzed via 2-way ANOVAs with sex and perinatal drug history as factors. While we would have liked to analyzed Figures 2 E & F in the same manner, these data depict progressive ratio which was conducted on different testing days in males and females due to females progressing through FR1 training in 4 fewer days (Figure 2 C & D). We refer the reviewer to the Statistics section for this explanation (Page 18 Line 389-394).

o Please make the Y-axes in Figures 2E and 2F the same scale.

RESPONSE: Completed.

• Figure 3 - Please make the Y-axes for Figures 3A through 2H the same scale.

RESPONSE: Thank you for catching this. To better observe male vs female performance, we have made each of the male & female outcomes on the same scale (2 A & E; 2 B & F; 2 C & G; 2 D & H). We hope this is acceptable, as % accuracy, % omissions, premature responses, and total trials are still presented with scales that accommodate the data range and each of these endpoints have dramatically different ranges.

• Figure 5

o Figure 5H

• Please add a figure legend to Figure 5H and add the appropriate significance mark within the legend.

RESPONSE: We apologize for the confusion and agree that the * in Figure 5 H above the male MO group was too small and looked like a data point. We have made this more clear in the revised Figure 5. We refer the reviewer to the Figure 5 legend where * indicates p < 0.05 vs. male SAL, 2-way ANOVA. The legend for Figure 5 H was intentionally omitted since it is redundant and to emphasize there is a lack of a main effect of perinatal drug exposure, consistent with the rest of our figures.

• Please explain if it’s necessary to use the “#” mark beside the title in Figure 5H. Could the single asterisk above the male morphine group be sufficient?

RESPONSE: Yes, we refer the reviewer to the Figure 5 legend where the # denotes p < 0.05 drug x sex interaction and the single asterisk above the male MO group indicates the result of the post hoc test.

• Figure 6 - Please provide methodological clarity. mPFC typically refers to medial PFC, not mid PFC. Did the authors separate mid PFC from caudal PFC? If this was the case, please include rationale for this choice.

RESPONSE: We agree that this was incorrect and unclear. We now consistently refer to medial PFC as mPFC and have rephrased that we separated mid medial PFC (m-mPFC) from caudal medial PFC (c-mPFC), given the observed rostral-caudal mPFC differences in motivated behaviors and cognitive control (Kouneiher et al., 2009)

Discussion

• Page 25 - Please clarify whether the males exposed to neonatal morphine exhibited reduced motivation or delayed learning?

RESPONSE: We have clarified that the male MO offspring had delayed learning “in FR1” and reduced motivation “in PR” to better illustrate our findings. (Page 26 Line 572)

• Please discuss whether children exposed to neonatal opioids demonstrate increased incidence of ADHD.

RESPONSE: We have added the following sentence: “Children exposed to opioids prenatally are more likely to exhibit attention deficit hyperactivity disorder symptoms and diagnoses (Azuine et al., 2019; Haugland et al., 2018).” (Page 27 Line 593-595)

REFERENCES

Azuine RE, Ji Y, Chang HY, Kim Y, Ji H, Dibari J, Hong X, Wang G, Singh GK, Pearson C, Zuckerman B, Surkan PJ, Wang X (2019) Prenatal Risk Factors and Perinatal and Postnatal Outcomes Associated with Maternal Opioid Exposure in an Urban, Low-Income, Multiethnic US Population. JAMA Netw Open.

Grecco GG, Atwood BK (2020) Prenatal opioid exposure enhances responsiveness to future drug reward and alters sensitivity to pain: A review of preclinical models and contributing mechanisms. eNeuro.

Haugland S, Fevang SKE, Bøe T, Nilsen SA, Sandtorv LB, Gjestad R, Elgen IB (2018) Symptoms Associated With Attention Deficit/Hyperactivity Disorder and Autism Spectrum Disorders in School-Aged Children Prenatally Exposed to Substances. Subst Abus Res Treat 12:117822181876577.

Hirai AH, Ko JY, Owens PL, Stocks C, Patrick SW (2021) Neonatal Abstinence Syndrome and Maternal Opioid-Related Diagnoses in the US, 2010-2017. JAMA - J Am Med Assoc 325:146-155.

Hol T, Niesink M, Van Ree JM, Spruijt BM (1996) Prenatal exposure to morphine affects juvenile play behavior and adult social behavior in rats. Pharmacol Biochem Behav 55:615-618.

Kopp BL, Wick D, Herman JP (2013) Differential effects of homotypic vs. heterotypic chronic stress regimens on microglial activation in the prefrontal cortex. Physiol Behav 122:246-252.

Kouneiher F, Charron S, Koechlin E (2009) Motivation and cognitive control in the human prefrontal cortex. Nat Neurosci 12:939-945.

Ramsey NF, Niesink RJM, Van Ree JM (1993) Prenatal exposure to morphine enhances cocaine and heroin self-administration in drug-naive rats. Drug Alcohol Depend 33:41-51.
