# Supplementary material for: Perinatal Morphine Exposure Leads to Sex-Dependent Executive Function Deficits and Microglial Changes in Mice
Source: eNeuro. 2022 Oct 13;9(5):ENEURO.0238-22.2022. doi: 10.1523/ENEURO.0238-22.2022 (PMC9581576; doi:10.1523/ENEURO.0238-22.2022)
Supplement: Figure 1-1 — End point, variables measured, offspring sample size, and litter representation for each outcome assessed. Download Figure 1-1, DOCX file. [file enu-eN-NWR-0238-22-s06.docx]

**Extended Data Figure 1-1:**

| **Endpoint** | **Variable** | **# Offspring per group** | **# Offspring per litter per group** |
| --- | --- | --- | --- |
| Pup retrieval | Latency to retrieve first pup, average retrieval latency per pup, percent of pups retrieved | SAL dams n =6  MO dams n = 9 | Entire litters |
| Three chambered social interaction test | Time spent investigating, frequency, and percent preference for 1.) mouse vs. empty cup and 2.) novel vs. familiar mouse | Male SAL n = 11  Male MO n = 11  Female SAL n = 10  Female MO n = 12 | 1-2 |
| Sucrose preference test | Percent preference on days 2 – 3 | Male SAL n = 12  Male MO n = 12  Female SAL n = 10  Female MO n = 12 | 1-2 |
| High fat preference test | Percent preference on days 2 – 3 | Male SAL n = 12  Male MO n = 12  Female SAL n = 7  Female MO n = 9 | 1-2 |
| Operant behavioral testing | Fixed ratio 1 (FR1) days to criterion, progressive ratio (PR) breakpoint, 5CSRTT premature responses, percent omitted, percent accuracy, total trials | Male SAL n = 11  Male MO n = 11  Female SAL n = 10  Female MO n = 12 | 1-2 |
| P21 gene expression | Fold change | Male SAL n = 6  Male MO n = 6  Female SAL n = 6  Female MO n = 6 | 1 |
| Adult gene expression | Fold change | Male SAL n = 11  Male MO n = 11  Female SAL n = 10  Female MO n = 12 | 1-2 |
| Iba1/CD68 Immunohistochemistry | 20x:  Whole frame cell count and integrated density per counted cell  40x:  Individual cell perimeter, area, and average integrated density within each cell | 20x:  Male SAL n = 5, Male MO n = 6, Female SAL n = 3, Female MO n = 5  40x:  Male SAL n = 4, Male MO n = 5, Female SAL n = 2, Female MO n = 3 | 1 |
