# Supplementary material for: Perinatal Morphine Exposure Leads to Sex-Dependent Executive Function Deficits and Microglial Changes in Mice
Source: eNeuro. 2022 Oct 13;9(5):ENEURO.0238-22.2022. doi: 10.1523/ENEURO.0238-22.2022 (PMC9581576; doi:10.1523/ENEURO.0238-22.2022)
Supplement: Figure 1-2 — Gene expression targets for P21 mPFC, AMG, VTA, and NAc. The + and – symbols indicate included (+) or omitted (–) targets for adult operant PFC. The ^ and ᵒ symbols indicate included (^) or omitted (ᵒ) targets for adult operant AMG. Download Figure 1-2, DOCX file. [file enu-eN-NWR-0238-22-s07.docx]

**Extended Data Figure 1-2:**

| **Gene name** | **Assay ID** | **Functional relevance** |
| --- | --- | --- |
| + ACTB ^ | Ref: 4352663 | Housekeeping gene |
| - C1QA ᵒ | Mm00432142_m1 | Complement C1q A chain: complement recognition, predominantly microglial ^1^ |
| + COMT ᵒ | Mm00514377_m1 | Catechol-O-Methyltransferase: catecholeamine degradation |
| + DLG4 (PSD95) ᵒ | Mm00492193_m1 | Discs Large MAGUK Scaffold Protein 4: postsynaptic density protein 95 |
| - DNMT1 ᵒ | Mm01151063_m1 | DNA Methyltransferase 1: maintains DNA methylation^2^ |
| + DNMT3A ᵒ | Mm00432881_m1 | DNA Methyltransferase 3 Alpha: de novo DNA methylation^2^ |
| - GAD1 ᵒ | Mm04207432_g1 | Glutamate Decarboxylase 1: catalyzes GABA production |
| - GADD45B ᵒ | Mm00435123_m1 | Growth Arrest And DNA Damage Inducible Beta: DNA demethylation^3^ |
| + GAPDH ᵒ | Ref: 4352661 | Housekeeping gene |
| - GPHN ᵒ | Mm00556895_m1 | Gephyrin: Anchors inhibitory neurotransmitter receptors (glycine and GABA) |
| - HDAC2 ᵒ | Mm00515108_m1 | Histone Deacetylase 2: downregulates histone acetylation, induces gene silencing and downregulation of target protein synthesis ^4^ |
| + ITGAM ^ | Mm01271250_m1 | Integrin Subunit Alpha M (CD11b; CR3): macrophage/microglial adhesion for phagocytosis |
| + LY96 (MD2) ^ | Mm01227593_m1 | Lymphocyte Antigen 96 (Myeloid Differentiation Factor 2): binds lipopolysaccharides (LPS) to TLR4, links receptor and signaling |
| + MBP ᵒ | Mm00521979_m1 | Myelin Basic Protein: Myelin formation and stabilization in CNS |
| - MECP2 ᵒ | Mm01193537_g1 | Methyl-CpG Binding Protein 2: chromatin regulation and development of neuronal networks^5^ |
| + MOG ᵒ | Mm01279062_m1 | Myelin Oligodendrocyte Glycoprotein: myelin completion and/or maintenance |
| + MYD88 ^ | Mm00440338_m1 | Myeloid Differentiation Primary Response Gene 88: TLR4 adapter protein |
| + OPRD1 ᵒ | Mm01180757_m1 | Opioid Receptor Delta 1 |
| + OPRK1 ᵒ | Mm01230885_m1 | Opioid Receptor Kappa 1 |
| + OPRM1 ᵒ | Mm01188089_m1 | Opioid Receptor Mu 1 |
| + PDYN ᵒ | Mm00457573_m1 | Opioid peptide formation |
| + PENK ᵒ | Mm01212875_m1 | Preproenkephalin |
| + PLP1 ᵒ | Mm01297210_m1 | Proteolipid Protein 1: myelin formation and stability |
| + PNOC ᵒ | Mm01314909_m1 | Prepronociceptin: ligand for OPRL1 |
| + PPIA ^ | Mm02342429_g1 | Housekeeping gene |
| - SETD7 ᵒ | Mm00499823_m1 | SET Domain Containing 7, Histone Lysine Methyltransferase: regulates DNMT1 activity^6^ |
| + SLC17A7 (VGLUT1) ^ | Mm00812886_m1 | Solute Carrier Family 17 Member 7 (Vesicular glutamate transporter 1): presynaptic glutamate uptake |
| - SLC6A3 (DAT) ᵒ | Mm00438388_m1 | Solute Carrier Family 6 Member 3 (Dopamine Transporter): dopamine clearance/reuptake from synapses |
| - SYP ᵒ | Mm00436850_m1 | Synaptophysin: neuronal synaptic vesicle glycoprotein |
| + TLR2 ᵒ | Mm0442346_m1 | Toll-like receptor 2: pathogen recognition for general pathogens |
| + TLR4 ^ | Mm00445273_m1 | Toll-like receptor 4: pathogen recognition receptor for lipopolysaccharide |
| + YWHAZ ^ | Mm03950126_s1 | Housekeeping gene |
