# Supplementary material for: Perinatal Morphine Exposure Leads to Sex-Dependent Executive Function Deficits and Microglial Changes in Mice
Source: eNeuro. 2022 Oct 13;9(5):ENEURO.0238-22.2022. doi: 10.1523/ENEURO.0238-22.2022 (PMC9581576; doi:10.1523/ENEURO.0238-22.2022)
Supplement: Figure 1-3 — Targets added for the adult operant mPFC gene expression analysis. The ^ and ᵒ symbols indicate included (^) or omitted (ᵒ) targets for adult operant AMG. Download Figure 1-3, DOCX file. [file enu-eN-NWR-0238-22-s08.docx]

**Extended Data Figure 1-3:**

| **Gene name** | **Assay ID** | **Functional relevance** |
| --- | --- | --- |
| ARC ᵒ | Mm01204954_g1 | Activity-Regulated Cytoskeleton-Associated Protein: plasticity protein |
| DDX3Y ᵒ | Mm00465349_m1 | DEAD-Box Helicase 3 Y-Linked: male-specific brain protein ^7^ |
| DRD2 ᵒ | Mm00438545_m1 | Dopamine receptor D2 |
| DRD3 ᵒ | Mm00432887_m1 | Dopamine receptor D3 |
| HSPA1A (HSP70) ᵒ | Mm01159846_s1 | Heat Shock Protein Family A (Hsp70) Member 1A: Stabilizes proteins and mediates folding of newly translated proteins |
| OPRL1 ᵒ | Mm00440563_m1 | Opioid Related Nociceptin Receptor 1 |
| PPP1R9B (SPINOPHILIN) ᵒ | Mm00552071_m1 | Protein Phosphatase 1 Regulatory Subunit 9B: scaffold protein for dendritic spines |
| PVALB ᵒ | Mm00443100_m1 | Parvalbumin: GABAergic interneuron marker |
| RELA (NFKB) ᵒ | Mm00501346_m1 | RELA Proto-Oncogene, NF-KB Subunit: signal transduction for inflammation |
| SLC17A6 (VGLUT2) ᵒ | Mm00499876_m1 | Solute Carrier Family 17 Member 6 (Vesicular glutamate transporter 2): presynaptic glutamate uptake |
