# Supplementary material for: Perinatal Morphine Exposure Leads to Sex-Dependent Executive Function Deficits and Microglial Changes in Mice
Source: eNeuro. 2022 Oct 13;9(5):ENEURO.0238-22.2022. doi: 10.1523/ENEURO.0238-22.2022 (PMC9581576; doi:10.1523/ENEURO.0238-22.2022)
Supplement: Figure 1-4 — Targets added for the adult operant AMG gene expression analysis. Download Figure 1-4, DOCX file. [file enu-eN-NWR-0238-22-s09.docx]

**Extended Data Figure 1-4:**

| **Gene name** | **Assay ID** | **Functional relevance** |
| --- | --- | --- |
| ADORA2A | Mm00802075_m1 | Adenosine A2a Receptor; upregulated in the AMG of susceptible offspring after maternal immune activation; GPCR involved in immune function ^8^ |
| CD68 | Mm03047343_m1 | Macrophage antigen CD68; lysosomal phagocytic marker |
| CDKN1A | Mm04205640_g1 | Cyclin dependent kinase inhibitor 1A; upregulated after morphine exposure^9^ |
| DRD1 | Mm01353211_m1 | Dopamine receptor D1 |
| ERBIN (ERBB2IP) | Mm01301876_m1 | Erbb2 Interacting Protein; maintains excitatory-inhibitory balance in the AMG^10^ |
| FKBP5 | Mm01300962_m1 | FKBP prolyl isomerase 5; stress response |
| GNG8 | Mm00515877_g1 | Guanine nucleotide-binding protein G(I)/G(S)/G(O) subunit gamma-8; downregulated in AMG after early life stress, involved in morphine addiction and synaptic function^11^ |
| GRIN1 | Mm00433790_m1 | Glutamate Ionotropic Receptor NMDA Type Subunit 1; synaptic plasticity |
| HES1 | Mm01342805_m1 | Hes Family BHLH Transcription Factor 1; involved in social behavior^12^ |
| HTR1A | Mm0043406_s1 | Serotonin Receptor 1A |
| HTR2A | Mm00555764_m1 | Serotonin Receptor 2A |
| IL1B | Mm00434228_m1 | Interleukin 1 beta; cytokine mediator of the inflammatory response |
| MAP4K1 | Mm01152700_m1 | Mitogen-Activated Protein Kinase Kinase Kinase Kinase 1; upregulated after early life stress^13^ |
| MAP4K2 | Mm01231599_m1 | Mitogen-Activated Protein Kinase Kinase Kinase Kinase 1; upregulated after early life stress^13^ |
| MAPK10 | Mm00436578_m1 | Mitogen-Activated Protein Kinase 10 |
| NP65 (NPTN) | Mm00485990_m1 | Neuroplastin 65; involved in synaptic plasticity and cognition^14^ |
| NR3C1 (GR) | Mm00433832_m1 | Nuclear Receptor Subfamily 3 Group C Member 1; glucocorticoid receptor |
| NTS | Mm00481140_m1 | Neurotensin; offspring sex-dependent expression changes after maternal immune activation^15^ |
| OXT | Mm01329577_m1 | Oxytocin |
| PPP1R1B | Mm00454892_m1 | DARPP-32; upregulated in the AMG of susceptible offspring after maternal immune activation^16^ |
| RELN | Mm00465200_m1 | Reelin; offspring sex-dependent expression changes after maternal immune activation^15^ |
| SGK1 | Mm00441380_m1 | Serum/Glucocorticoid Regulated Kinase 1; upregulated after morphine exposure^9^ |
| TLR7 | Mm00446590_m1 | Toll Like Receptor 7 |
