# Supplementary figures and images for: Perinatal Morphine Exposure Leads to Sex-Dependent Executive Function Deficits and Microglial Changes in Mice
Source: eNeuro. 2022 Oct 13;9(5):ENEURO.0238-22.2022. doi: 10.1523/ENEURO.0238-22.2022 (PMC9581576; doi:10.1523/ENEURO.0238-22.2022)

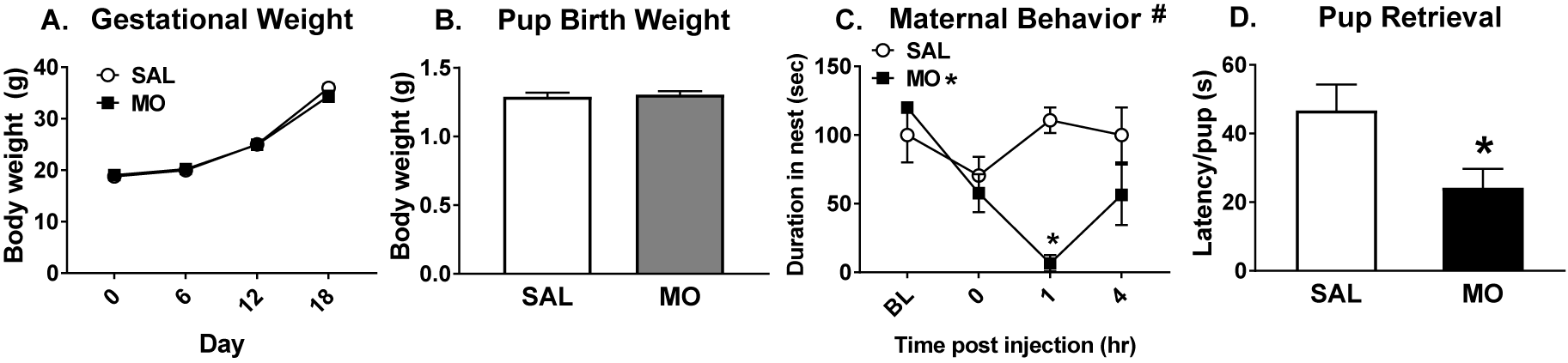

Supplement: Figure 1-5 — Maternal and birth characteristics. A, B, Maternal MO treatment did not alter gestational weight (A) or pup birth weight (B). C, Assessed during the first postnatal week, MO injection acutely reduced time spent in the nest at 1 h, but this was recovered by 4 h (#p < 0.05, drug × time interaction; MO: *p < 0.05 main effect of drug; *p < 0.05 MO vs SAL at 1 h, two-way repeated-measures ANOVA). D, On postnatal day 2, MO dams returned their pups faster on average than SAL dams (*p < 0.05 vs SAL, t test). Download Figure 1-5, TIF file. [file enu-eN-NWR-0238-22-s10.tif]

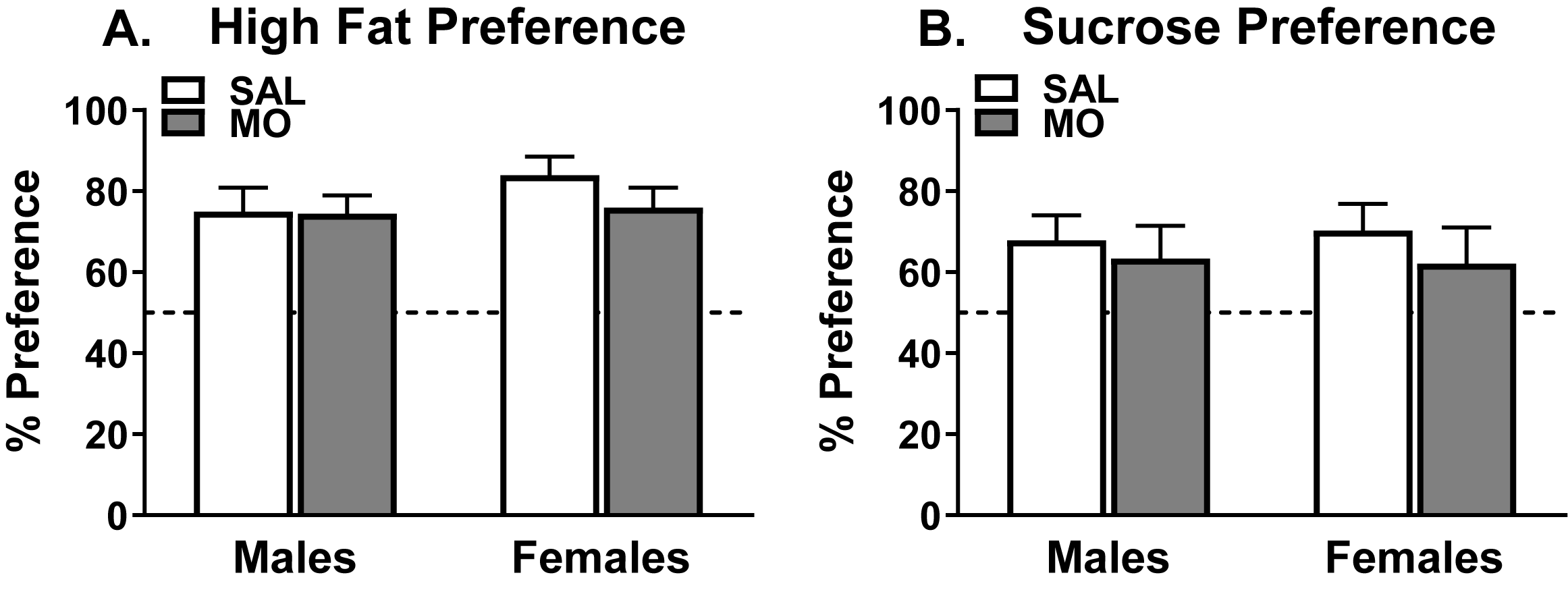

Supplement: Figure 2-1 — High-fat and sucrose preference tests. A, B, There were no group differences in high-fat (A) or sucrose preference (B) tests. Download Figure 2-1, TIF file. [file enu-eN-NWR-0238-22-s04.tif]

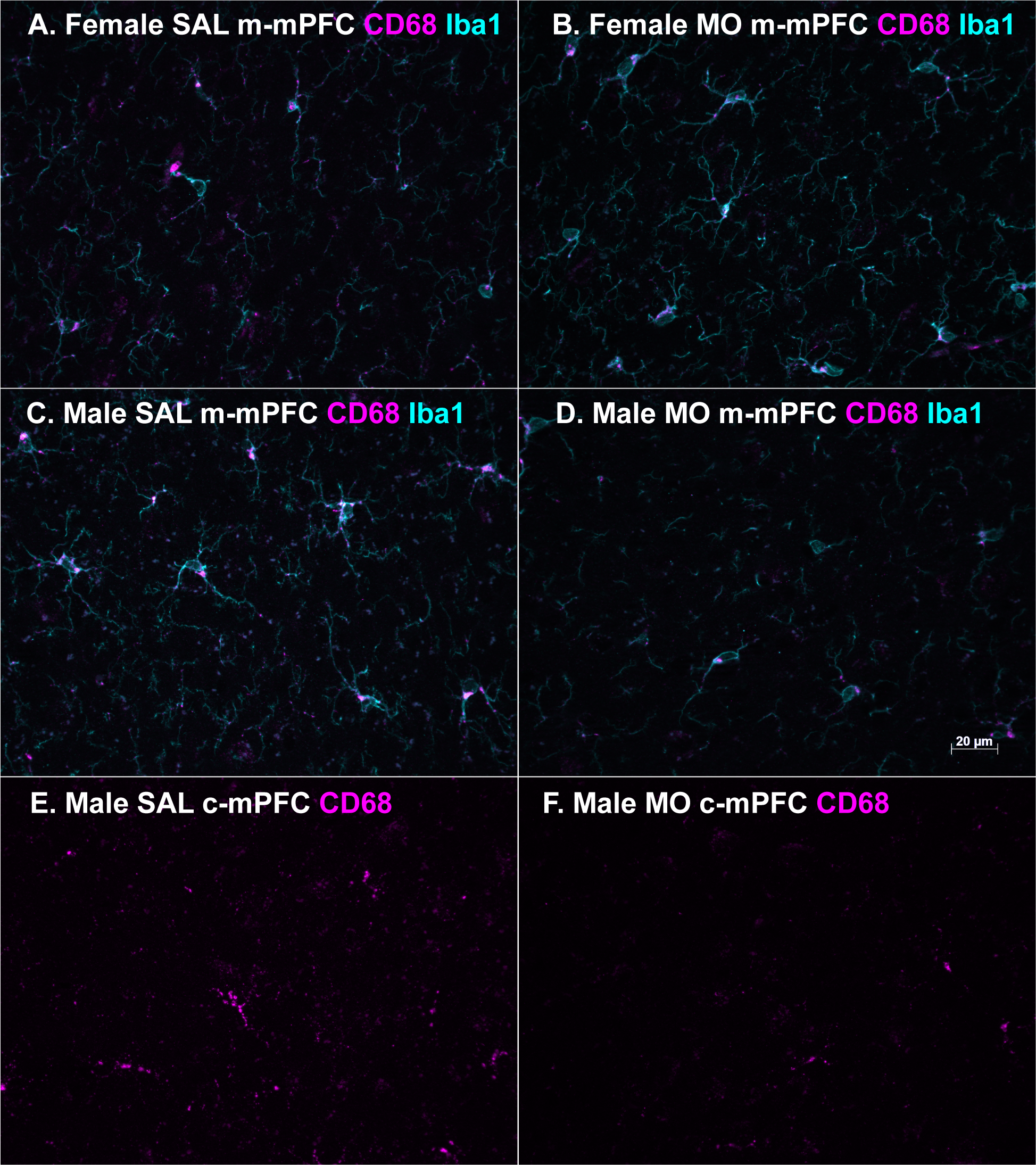

Supplement: Figure 6-2 — Representative images for CD68/Iba1 immunohistochemistry. A–D, Representative m-mPFC Iba1/CD68 40× images of female SAL (A) and female MO (B) depict increased Iba1 in female MO offspring; male SAL (C) and male MO (D) depict reduced Iba1 in male MO offspring. Representative c-mPFC CD68 40× images of male SAL (L) and male MO (O) depict reduced CD68 in male MO offspring. Download Figure 6-2, TIF file. [file enu-eN-NWR-0238-22-s05.tif]
