# Supplementary material for: Perinatal Morphine Exposure Leads to Sex-Dependent Executive Function Deficits and Microglial Changes in Mice
Source: eNeuro. 2022 Oct 13;9(5):ENEURO.0238-22.2022. doi: 10.1523/ENEURO.0238-22.2022 (PMC9581576; doi:10.1523/ENEURO.0238-22.2022)
Supplement: Figure 1-6 — Values for maternal and birthing outcomes that did not reach statistical significance (mean ± SEM) or median (range). Download Figure 1-6, DOCX file. [file enu-eN-NWR-0238-22-s11.docx]

**Extended Data Figure 1-6:**

| **Outcome** | **Maternal SAL** | **Maternal MO** | **Result** |
| --- | --- | --- | --- |
| Gestational duration from plugs (days) | 17.9 ± 0.26 | 18.3 ± 0.36 | [T_(12)_ = 0.96; p = 0.35] |
| Litter size (pups) | 8.7 ± 0.42 | 6.4 ± 1.11 | [T_(14)_ = 0.96; p = 0.35] |
| Maternal nest volume (cm^3^) | 340.7 (226.4 – 954.2) | 222.7 (171.3 – 469.4) | [U_(1, 13)_ = 9; p = 0.10] |
