# Supplementary material for: Perinatal Morphine Exposure Leads to Sex-Dependent Executive Function Deficits and Microglial Changes in Mice
Source: eNeuro. 2022 Oct 13;9(5):ENEURO.0238-22.2022. doi: 10.1523/ENEURO.0238-22.2022 (PMC9581576; doi:10.1523/ENEURO.0238-22.2022)
Supplement: Figure 1-7 — Values for offspring body weight (mean ± SEM). Download Figure 1-7, DOCX file. [file enu-eN-NWR-0238-22-s12.docx]

**Extended Data Figure 1-7:**

| **Outcome** | **Male**  **SAL** | **Mal**  **MO** | **Female SAL** | **Female**  **MO** | **Result** |
| --- | --- | --- | --- | --- | --- |
| Offspring body weight P21 | 9.1 ± 0.25 | 9.3 ± 0.37 | 8.7 ± 0.16 | 9.2 ± 0.32 | Interaction [F_(1, 22)_ = 0.3; p = 0.59]  Sex [F_(1, 22)_ = 0.5; p = 0.48]  Drug [F_(1, 22)_ = 1.7; p = 0.21] |
| Offspring body weight P42 | 21.7 ± 0.27 | 21.8 ± 0.56 | 17.4 ± 0.27 | 17.6 ± 0.19 | Interaction [F_(1, 42)_ = 0.04; p = 0.84]  **Sex [F_(1, 42)_ = 142.9; p < 0.0001]**  Drug [F_(1, 42)_ = 0.2; p = 0.65] |
