# Supplementary material for: Perinatal Morphine Exposure Leads to Sex-Dependent Executive Function Deficits and Microglial Changes in Mice
Source: eNeuro. 2022 Oct 13;9(5):ENEURO.0238-22.2022. doi: 10.1523/ENEURO.0238-22.2022 (PMC9581576; doi:10.1523/ENEURO.0238-22.2022)
Supplement: Figure 5-1 — P21 fold change values by group (mean ± SEM) for observed sex differences in gene expression. Download Figure 5-1, DOCX file. [file enu-eN-NWR-0238-22-s02.docx]

**Extended Data Figure 5-1:**

| **Brain Region, Gene Name** | **Male**  **SAL** | **Mal**  **MO** | **Female SAL** | **Female**  **MO** | **Sex Effect** |
| --- | --- | --- | --- | --- | --- |
| AMG DLG4 | 1.01 ± 0.06 | 0.90 ± 0.03 | 1.06 ± 0.04 | 1.05 ± 0.04 | [F_(1, 19)_ = 5.2; p = 0.034] |
| AMG DNMT1 | 1.00 ± 0.04 | 0.99 ± 0.03 | 1.15 ± 0.05 | 1.03 ± 0.03 | [F_(1, 19)_ = 6.6; p = 0.019] |
| AMG ITGAM | 1.01 ± 0.06 | 1.00 ± 0.02 | 1.15 ± 0.06 | 1.08 ± 0.03 | [F_(1, 19)_ = 5.8; p = 0.026] |
| AMG MBP | 1.00 ± 0.05 | 0.91 ± 0.04 | 1.15 ± 0.09 | 1.04 ± 0.04 | [F_(1, 19)_ = 7.2; p = 0.015] |
| AMG SETD7 | 1.00 ± 0.04 | 1.01 ± 0.02 | 1.13 ± 0.04 | 1.08 ± 0.05 | [F_(1, 19)_ = 6.2; p = 0.022] |
| AMG OPRK1 | 1.01 ± 0.08 | 1.17 ± 0.05 | 0.87 ± 0.06 | 0.92 ± 0.13 | [F_(1, 19)_ = 5.2; p = 0.035] |
| NAC MYD88 | 1.00 ± 0.02 | 0.99 ± 0.04 | 1.09 ± 0.08 | 1.12 ± 0.03 | [F_(1, 20)_ = 6.7; p = 0.017] |
| NAC TLR2 | 1.00 ± 0.05 | 1.00 ± 0.06 | 1.13 ± 0.06 | 1.12 ± 0.06 | [F_(1, 20)_ = 5.1; p = 0.035] |
