# Supplementary material for: Perinatal Morphine Exposure Leads to Sex-Dependent Executive Function Deficits and Microglial Changes in Mice
Source: eNeuro. 2022 Oct 13;9(5):ENEURO.0238-22.2022. doi: 10.1523/ENEURO.0238-22.2022 (PMC9581576; doi:10.1523/ENEURO.0238-22.2022)
Supplement: Figure 6-1 — Male m-mPFC values for Iba1 and CD68 reported as the mean ± SEM. Download Figure 6-1, DOCX file. [file enu-eN-NWR-0238-22-s03.docx]

**Extended Data Figure 6-1:**

| **Outcome** | **Male**  **SAL** | **Mal**  **MO** | **Result** |
| --- | --- | --- | --- |
| Adult male mid mPFC 20x Iba1 | 13520234 ± 1744167 | 13343172 ± 1394147 | [T_(9)_ = 0.08; p = 0.94] |
| Adult male mid mPFC 20x CD68 | 5491359 ± 770503 | 6188160 ± 1078053 | [T_(9)_ = 0.5; p = 0.62] |
